# *MAP3K1* mutations confer tumor immune heterogeneity in hormone receptor–positive HER2-negative breast cancer

**DOI:** 10.1172/JCI183656

**Published:** 2024-11-12

**Authors:** Yu-Wen Cai, Cui-Cui Liu, Yan-Wu Zhang, Yi-Ming Liu, Lie Chen, Xin Xiong, Zhi-Ming Shao, Ke-Da Yu

**Affiliations:** 1 Department of Breast Surgery, Fudan University Shanghai Cancer Center and Cancer Institute, Department of Oncology, Shanghai Medical College, Fudan University, Shanghai, China.; 2 Key Laboratory of Breast Cancer in Shanghai, Shanghai, China.; 3 Department of Breast Surgery, The Third Affiliated Hospital of Zhengzhou University, Zhengzhou, China.

**Keywords:** Immunology, Oncology, Cancer, Immunotherapy

## Abstract

Treatment for hormone receptor–positive/human epidermal growth factor receptor 2–negative (HR^+^/HER2^−^) breast cancer, the most common type of breast cancer, has faced challenges such as endocrine therapy resistance and distant relapse. Immunotherapy has shown progress in treating triple-negative breast cancer, but immunological research on HR^+^/HER2^–^ breast cancer is still in its early stages. Here, we performed a multi-omics analysis of a large cohort of patients with HR^+^/HER2^–^ breast cancer (*n* = 351) and revealed that HR^+^/HER2^–^ breast cancer possessed a highly heterogeneous tumor immune microenvironment. Notably, the immunological heterogeneity of HR^+^/HER2^–^ breast cancer was related to mitogen-activated protein kinase kinase kinase 1 (*MAP3K1*) mutation and we validated experimentally that a *MAP3K1* mutation could attenuate CD8^+^ T cell–mediated antitumor immunity. Mechanistically, *MAP3K1* mutation suppressed MHC-I–mediated tumor antigen presentation through promoting the degradation of *antigen peptide transporter 1/2* (*TAP1*/*2*) mRNA, thereby driving tumor immune escape. In preclinical models, the postbiotic tyramine could reverse the *MAP3K1* mutation–induced MHC-I reduction, thereby augmenting the efficacy of immunotherapy. Collectively, our study identified the vital biomarker driving the immunological heterogeneity of HR^+^/HER2^–^ breast cancer and elucidated the underlying molecular mechanisms, which provided the promise of tyramine as what we believe to be a novel therapeutic strategy to enhance the efficacy of immunotherapy.

## Abstract 2

<strong>*MAP3K1* mutations drive immune escape in Hormone receptor-positive and HER2-negative breast cancer by reducing MHCI-mediated tumor antigen presentation</strong>

## Introduction

Breast cancer is the most common cancer worldwide in female patients. Hormone receptor–positive (HR^+^, defined as positive estrogen receptor [ER] and/or positive progesterone receptor [PR] status) and human epidermal growth factor receptor 2-negative (HER2^−^) breast cancer accounts for 65%–75% of all breast cancers, for which endocrine therapy is the standard of care (1). Although HR^+^/HER2^–^ breast cancer is highly endocrine responsive, resistance to endocrine therapy is common (2), and the risk of recurrence and death from breast cancer persists for over 20 years after original diagnosis (3). New approaches, including novel therapies and combination treatments, are therefore urgently needed to refine and improve the treatment strategies for patients.

Over the past few years, immunotherapy has shown great promise in multiple tumor types, such as melanoma, non-small cell lung cancer and triple-negative breast cancer (TNBC). Immune checkpoint inhibitors (ICIs) have benefited patients with TNBC, with the relevant fundamental research and clinical transformation being well established. However, the therapeutic exploration of ICIs in HR^+^/HER2^–^ breast cancer is still in its early stages. Phase I–II clinical studies with small sample size, such as GIADA (NCT04659551) (4), KEYNOTE-028 (NCT02054806) (5), NCT02779751 (6), NCT03051659 (7), and more (8), provided the preliminary efficacy and safety data of ICIs in HR^+^/HER2^–^ breast cancer. The I-SPY2 study suggested that there was a significantly higher pathologic complete response (pCR) rate in patients who received ICI treatment than those who received neoadjuvant chemotherapy alone (9, 10). Recent large phase III clinical studies, KEYNOTE-756 (NCT03725059, *n* = 1,278) and CheckMate-7FL (NCT04109066, *n* = 510), showed that the pCR rate in the ICI treatment group was significantly improved compared with the pCR rate in the control group, which confirmed the prospect of immunotherapy in treating HR^+^/HER2^–^ breast cancer. Notably, the pCR rate is generally 20% in HR^+^/HER2^–^ breast cancer, which is far less than the 50% rate seen in TNBC. Moreover, the subgroup analyses in the 2 large trials indicated that pCR rate varied from 5% to 50% in different subgroups, suggesting that HR^+^/HER2^–^ breast cancer possessed a highly heterogeneous tumor immune microenvironment. Therefore, we need to identify the key biomarkers of immune heterogeneity and further dissect the biological mechanisms underlying the immunological resistance in order to develop novel, tailored treatment strategies to enhance immunotherapy efficacy in treating HR^+^/HER2^–^ breast cancer.

Here, we performed a comprehensive analysis of the clinical, genomic, and transcriptomic data of HR^+^/HER2^–^ breast cancers (*n* = 351) in the Chinese Breast Cancer Genome Atlas (CBCGA) cohort, which we previously reported (11) and revealed that there were 2 immunologically heterogeneous phenotypes. Notably, the immunosuppressive phenotype was associated with a high prevalence of mitogen-activated protein kinase kinase kinase 1 (*MAP3K1*) mutation, which suppressed MHC-I–mediated tumor antigen presentation by promoting the degradation of *antigen peptide transporter 1/2* (*TAP1/2*) mRNAs, and thus reshaped the tumor immune microenvironment of HR^+^/HER2^–^ breast cancers.

## Results

### MAP3K1 mutation is closely correlated with immune microenvironment heterogeneity in HR^+^/HER2^–^ breast cancer.

**Figure 1:**
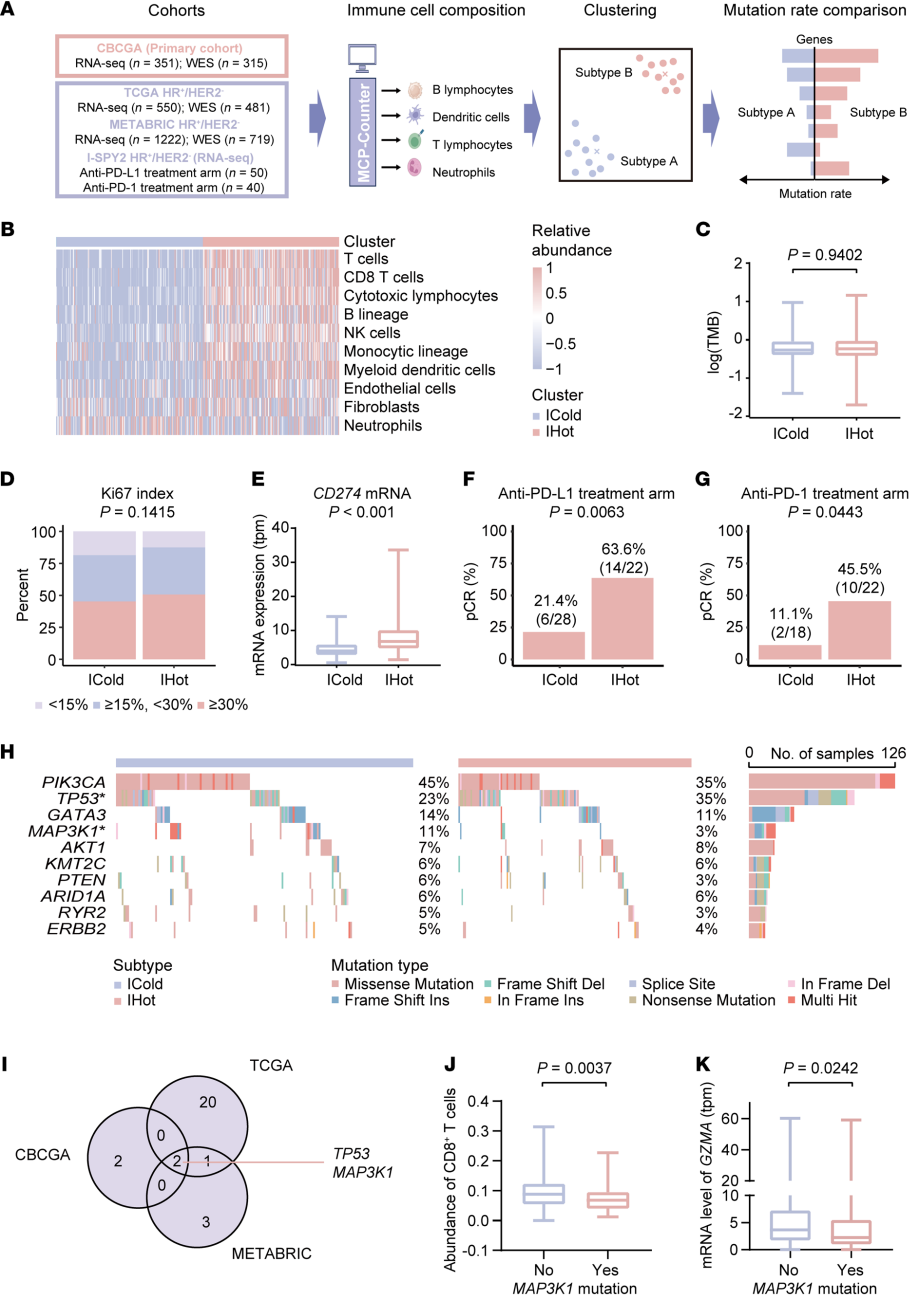
*MAP3K1* mutation is closely correlated with immune microenvironment heterogeneity in HR^+^/HER2^–^ breast cancer. (**A**) Flowchart of bioinformatics analyses performed in the study. (**B**) Heatmap showing the relative abundance of immune and stromal cells calculated by MCP-Counter in each sample in CBCGA cohort (*n* = 351). The 2 immunological subtypes were annotated. (**C**) Comparison of tumor mutation burden (TMB) of tumors with the ICold and IHot subtypes in CBCGA cohort (*n* = 314). The center line represents the median. (**D** and **E**) Comparison of ki67 index (**D**) and *CD274* mRNA expression level (**E**) of tumors with the ICold and IHot subtypes in CBCGA cohort (*n* = 350). The center line represents the median. (**F** and **G**) Pathological complete response (pCR) rate of patients with the ICold and IHot subtypes in the anti-PD-L1 (**F**) and anti-PD-1 (**G**) treatment arm of the I-SPY2 clinical trial. (**H**) The somatic mutations identified in tumors with the ICold and IHot subtypes in CBCGA cohort (*n* = 314). *P < 0.05. (**I**) Venn diagram showing genes with significantly different mutation prevalence between the ICold and IHot subtypes in CBCGA (*n* = 314), TCGA HR^+^/HER2^–^ (*n* = 475), and METABRIC HR^+^/HER2^–^ (*n* = 611) breast cancer cohorts. (**J** and **K**) Abundance of CD8^+^ T cells calculated by CIBERSORT (**J**) and *GZMA* mRNA expression (**K**) in HR^+^/HER2^–^ breast tumors with or without *MAP3K1* mutation in the TCGA cohort (*n* = 481). Statistical analysis: (**C**, **E**, **J**, and **K**) Wilcoxon signed-rank test; (**D**) χ^2^ test for trend; (**F**–**H**) Fisher’s exact test. ICold, immune cold subtype; IHot, immune hot subtype; TMB, tumor mutation burden; HR, hormone receptor; HER2, human epidermal growth factor receptor 2.

To explore the heterogeneity of the HR^+^/HER2^–^ breast cancer immune microenvironment, we performed multi-omics analysis with our CBCGA cohort (Figure 1A). First, we employed the microenvironment cell populations-counter (MCP-counter) analysis to depict the microenvironment features of HR^+^/HER2^–^ breast tumors. The results of NbClust analysis showed that 2 was the optimal and stable clustering number (Supplemental Figure 1A; supplemental material available online with this article; https://doi.org/10.1172/JCI183656DS1). Then, we performed the partitioning around medoid (PAM) clustering analysis and classified the HR^+^/HER2^–^ breast cancers into 2 subtypes, namely immune-hot (IHot, characterized by a relatively high immune cell and low stromal cell infiltration level) and immune-cold (ICold, characterized by a converse landscape) (Figure 1B and Supplemental Figure 1B). For the ICold subtype, we considered that the low infiltration level of immune cells in partial tumors was attributed to the relatively benign nature of the cancer, which is associated with a good prognosis in clinic. We therefore discarded patients who had had a followup time of over 10 years and continued to survive with the ICold subtype (Supplemental Table 1). The remaining patients with the ICold subtype were subjected to further analysis.

As expected, the tumor mutation burden (TMB) and ki-67 index/*MKI67* mRNA expression in the ICold subtype were comparable to or higher than what was seen in the IHot subtype (Figure 1, C and D and Supplemental Figure 1, C and D). However, there was a lower *CD274* (the gene encoding PD-L1) mRNA expression in the ICold than IHot subtype (Figure 1E and Supplemental Figure 1E). To further explore whether they have varying response to immunotherapy, we then extrapolated the 2 subtypes to I-SPY2 (9, 10), a neoadjuvant platform trial, and discovered that, whether receiving anti-PD-L1 or anti-programmed death-1 (PD-1) treatment, the ICold subtype achieved a significantly lower pCR rate than the IHot subtype (Figure 1, F and G). In summary, we revealed 2 immunologically distinct subtypes in terms of immune infiltration, PD-L1 expression, and response to immunotherapy with comparable TMB and clinical features, indicating that HR^+^/HER2^–^ breast cancer possessed a highly heterogeneous immune environment.

We further explored the pivotal biomarkers contributing to the immune microenvironment heterogeneity in HR^+^/HER2^–^ breast cancers. We depicted the nonsynonymous somatic mutation landscape of tumors in the CBCGA cohort (Figure 1H) and compared the mutation prevalence of cancer-related genes between the ICold and IHot subtypes in CBCGA, TCGA, and METABRIC cohorts (Supplemental Figure 1F). The intersection of the results from 3 cohorts revealed 2 candidate genes: *TP53* and *MAP3K1* (Figure 1I). Previous studies have demonstrated the important role of p53 in regulating antitumor immunity (12–14), which confirmed the reliability of our analyses. MEKK1, the gene product of *MAP3K1*, is a 196-kDa serine-threonine kinase in the MAPK family with functions in cell viability, apoptosis, and cell motility/migration in multiple normal and tumor cell types. To our knowledge, it has not been reported that there was an association between *MAP3K1* mutations and the efficacy of antitumor immunotherapy, which triggered our interest. To further explore the function of *MAP3K1* mutation on regulating tumor immune microenvironment, we performed the cell-type identification by estimating relative subsets of RNA transcripts (CIBERSORT) analysis and discovered that tumors with *MAP3K1* mutation had a lower abundance of CD8^+^ T cells, the main immune effector cells for antitumor immunity, compared with tumors without *MAP3K1* mutation (Figure 1J). Moreover, tumors with *MAP3K1* mutation also had a significantly lower mRNA expression of *GZMA*, a cytotoxicity marker of T cells (Figure 1K). These results indicated that the *MAP3K1* gene was closely correlated with immune heterogeneity in HR^+^/HER2^–^ breast cancer and its mutation could induce an immunosuppressive microenvironment.

### Map3k1-mutant tumors evade CD8^+^ T cell–mediated immunity in vivo.

**Figure 2:**
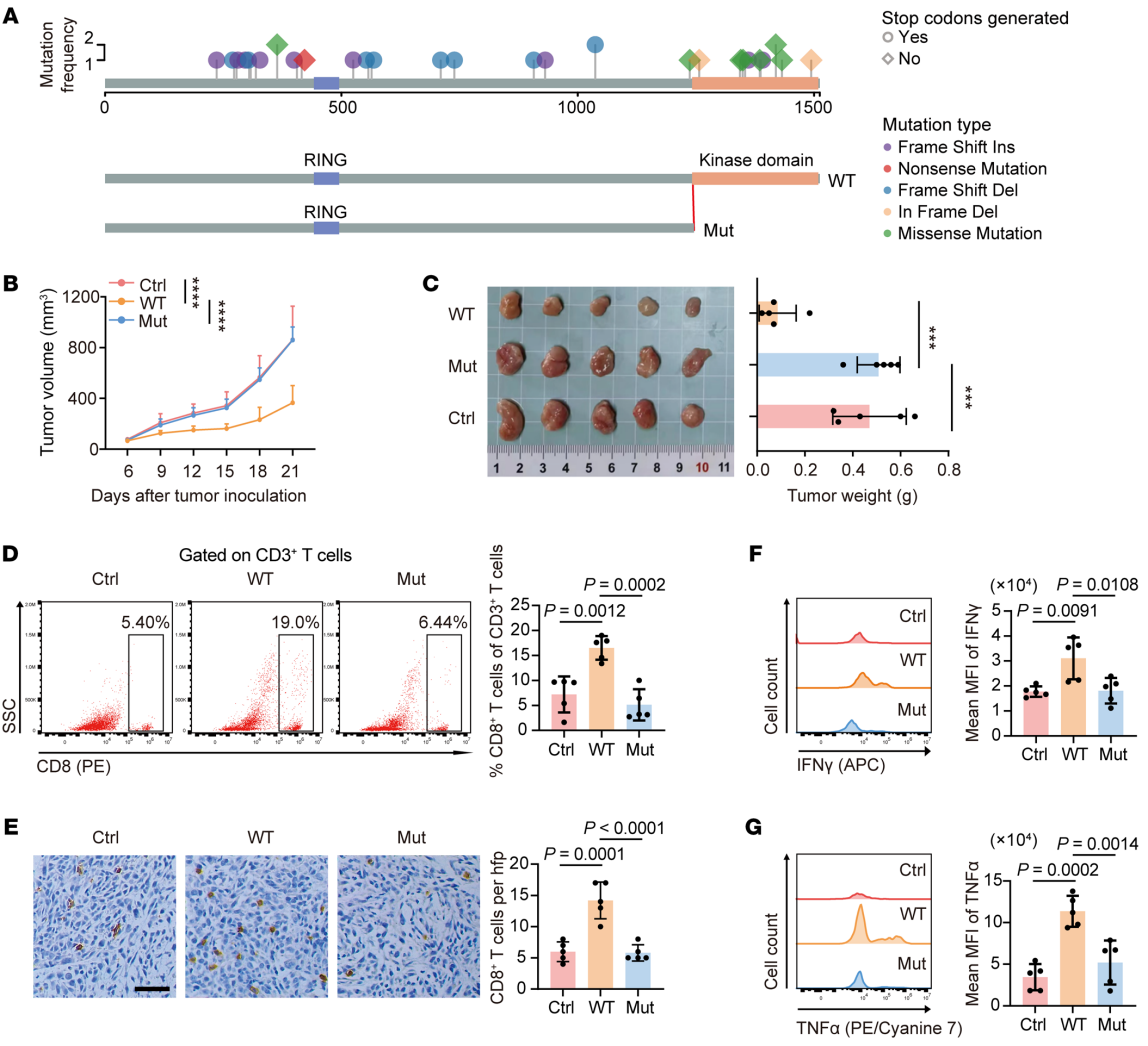
*Map3k1*-mutant tumors evade CD8^+^ T cell–mediated immunity in vivo. (**A**) *MAP3K1* mutation atlas of tumors in the CBCGA cohort and schematic diagram of full-length (WT) and kinase domain-truncated (1–1,222 aa) *Map3k1* (Mut) overexpression plasmids generated for the following experiments. The mutation type and whether a stop codon was generated are annotated. (**B** and **C**) 67NR mouse breast cancer cells with varying *Map3k1* status (overexpression based on *Map3k1*-KO cell lines) were orthotopically injected into BALB/c mice (*n* = 5 per group). Tumor growth curves (**B**) and tumor weights with the images (**C**) are shown. (**D**) Representative flow cytometry data of CD8^+^ T cell infiltration gated on CD3^+^ T cells in tumor tissues. (**E**) Representative IHC images of tumor tissues are shown and the numbers of CD8^+^ T cell are quantified. Scale bar, 50 μm. (**F** and **G**) MFI of IFN-γ (**F**) and TNF-α (**G**) in CD8^+^ T cells in the tumor tissues. Data are mean ± SD (**B**–**G**) (*n* = 5 per group). Statistical analysis: (**B**) 2-way ANOVA with Tukey’s test; (**C**–**G**) 1-way ANOVA with Tukey’s test. Significance in tumor growth (**B**) and tumor weight (**C**). *P < 0.05, **P < 0.01, ***P < 0.001, ****P < 0.0001.

MEKK1, the gene product of *MAP3K1*, consists of a RING zinc finger domain near the N-terminus and a serine/threonine kinase domain at the C-terminus. We observed that a majority of *MAP3K1* mutation in the patient cohort was truncated, resulting the loss of the kinase domain of MEKK1 (Figure 2A). To further investigate the effect of *MAP3K1* mutation on tumor progression, we knocked out its endogenous expression and then overexpressed either *Map3k1* WT or C-terminus kinase domain-truncated mutant (mut) in murine luminal breast cancer cell lines 67NR and EMT6. The KO efficacy (Supplemental Figure 2A) and overexpression efficacy (Supplemental Figure 2B) were confirmed by Western blot.

We first assessed the effect of *Map3k1* gene itself on cancer cells and discovered that *Map3k1*-WT significantly promoted cell proliferation in vitro, which is consistent with previously reported researches (15–17), while *Map3k1*-mut abolished this promoting effect (Supplemental Figure 2C). Data from the METABRIC cohort also indicated a lower *MKI67* mRNA expression in tumors with *MAP3K1* mutation (Supplemental Figure 2D). With the above mutation model, we also employed an orthotopic mammary xenograft model in BALB/c mice. Interestingly, *Map3k1*-WT inhibited the tumor growth but *Map3k1*-Mut displayed the opposite effect on tumor growth in vivo (Figure 2, B and C and Supplemental Figure 2, E and F). These results indicated that the tumor immune microenvironment could be responsible for the inconsistency of the effect of *Map3k1* mutation status in vivo and in vitro.

To validate our speculation, we isolated tumors from mice and analyzed the microenvironment characteristics. As expected, we found a reduced proportion and inhibited function of CD8^+^ T cells in *Map3k1*-mut tumors (Figure 2, D–G and Supplemental Figure 2, G–J), which might explain the faster tumor progression of *Map3k1*-mut tumors compared with *Map3k1*-WT tumors in vivo. To further confirm the necessity of CD8^+^ T cell–mediated tumor immunity in *Map3k1*-modulated tumor growth, we used a CD8 neutralizing antibody to deplete CD8^+^ T cells in mice (Supplemental Figure 3, A and B). We observed that depletion of CD8^+^ T cells abolished the growth difference among tumors with varying *Map3k1* mutation status (Supplemental Figure 3, C and D), indicating the critical role of CD8^+^ T cells in mediating the effects of *Map3k1* on tumor growth in vivo.

### Map3k1-mutant tumor cells suppress CD8^+^ T cell–mediated immunity in vitro.

**Figure 3:**
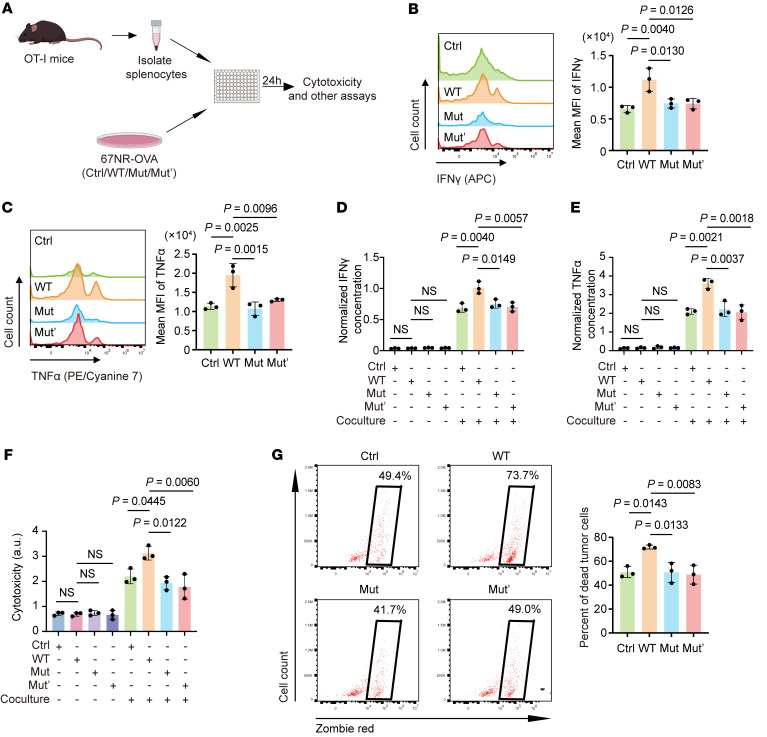
*MAP3K1* mutation inhibits CD8^+^ T cell activation in vitro. (**A**) Schematic diagram of the in vitro coculture assay. OVA-expressing 67NR tumor cells with varying *Map3k1* status were cocultured with splenocytes isolated from OT-I mice at a ratio of 1:10. At 24 hours after coculture, tumor cells, immune cells, and the culture medium were collected for the following analyses. (**B** and **C**) MFI of IFN-γ (**B**) and TNF-α (**C**) in CD8^+^ T cells are shown. (**D** and **E**) Concentration of cytokines IFN-γ (**D**) and TNF-α (**E**) in the culture medium was measured by ELISA. (**F**) T cell cytotoxicity was measured by lactate dehydrogenase (LDH) assay. (**G**) T cell cytotoxicity was assessed by the percentage of dead cells in EpCAM^+^ tumor cells, which are indicated by black boxes. Data are mean ± SD (**B**–**G**) (*n* = 3 per group). Statistical analysis: (**B**–**G**) 1-way ANOVA with Tukey’s test.

To test whether *Map3k1* status in tumor cells can directly impact the function of CD8^+^ T cells in vitro, we generated 67NR and EMT6 cell lines with varying *Map3k1* status that stably present a chicken ovalbumin peptide (OVA_257-264_). We then constructed an in vitro coculture system by coculturing these OVA-expressing cell lines and splenocytes isolated from an OT-I transgenic mouse, the CD8^+^ T cells of which can specifically recognize the OVA_257-264_ antigen in the context of MHC-I allele H-2K^b^ (18), and thus elicit strong epitope-specific immune responses (Figure 3A). To confirm the reliability of our findings regarding the function of *Map3k1* mutation, we also constructed another high-frequency mutant (Mut’, MEKK1-K1037R^fs*4^ mutant, which possessed high mutation rate in the CBCGA cohort) for the vitro experiments.

In the coculture system, we discovered a significantly reduced proportion of cytokine IFN-γ–positive CD8^+^ and tumor necrosis factor-α–positive (TNF-α–positive) CD8^+^ T cells (Figure 3, B and C and Supplemental Figure 4, A and B), consistent with a markedly reduced levels of cytokines IFN-γ and TNF-α in *Map3k1*-mut/mut’ groups compared with the *Map3k1*-WT group (Figure 3, D and E and Supplemental Figure 4, C and D). The reduced cytotoxicity of CD8^+^ T cells in the coculture system induced by *Map3k1*-mut/mut’ tumor cells was confirmed by lactate dehydrogenase (LDH) assay (Figure 3F and Supplemental Figure 4E). In line with these findings, we also discovered that *Map3k1*-mut/mut’ tumor cells had a significantly lower percentage of dead tumor cells in the coculture system (Figure 3G and Supplemental Figure 4F). Overall, these findings suggest that *Map3k1*-mut/mut’ tumor cells have an enhanced ability to evade killing by CD8^+^ T cells in vitro.

### Map3k1 mutation inhibits tumor antigen presentation.

**Figure 4:**
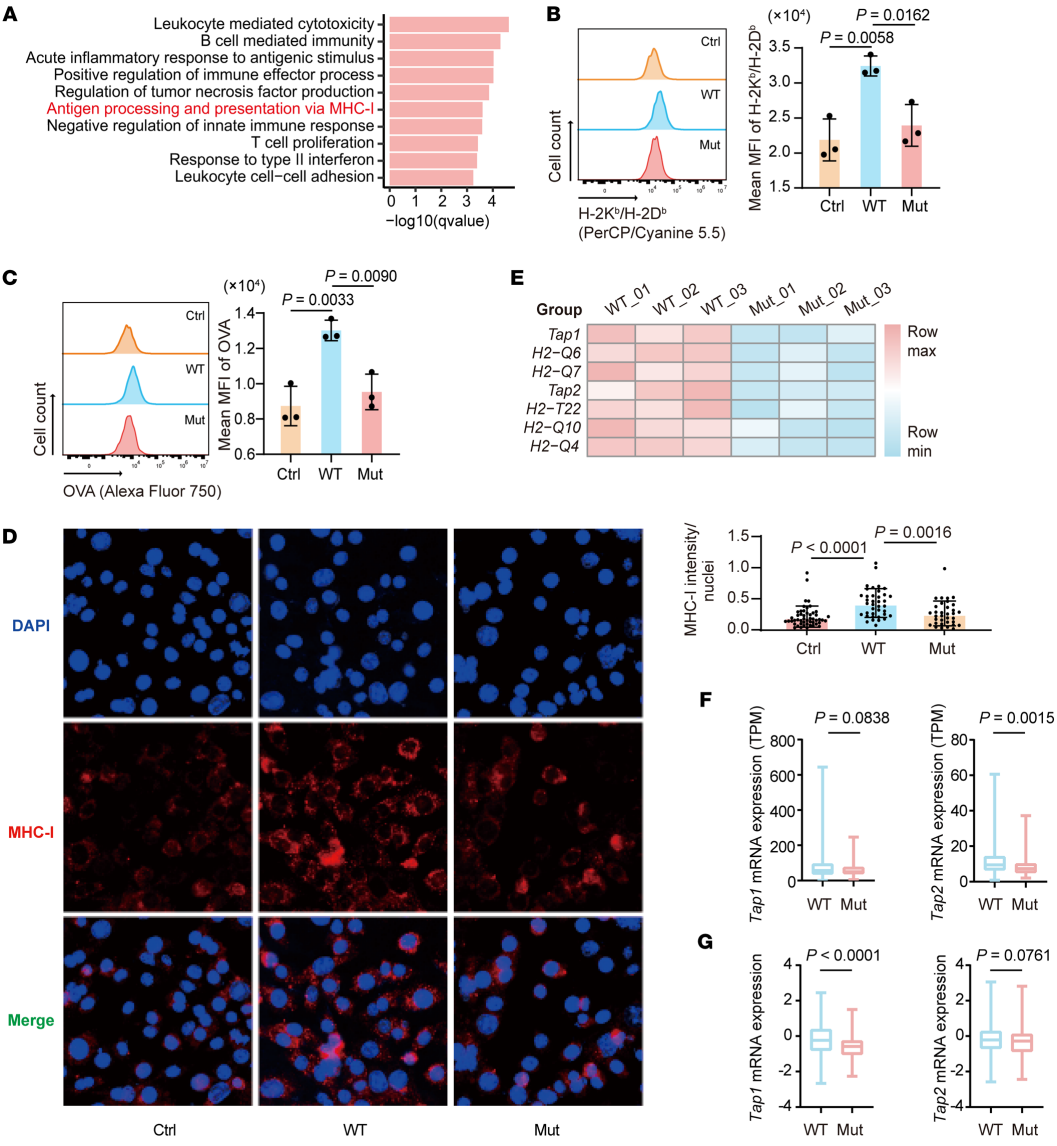
*MAP3K1* mutation suppresses tumor antigen presentation. (**A**) *Map3k1*-WT and *Map3k1*-mut tumor tissues were collected for RNA-seq. GO enrichment analysis was performed and immune-related pathways that are significantly downregulated in *Map3k1*-mut compared with *Map3k1*-WT tumors are shown here. (*n* = 3 per group). (**B** and **C**) Surface expression of H-2K^b^/H-2D^b^ (**B**) and OVA (**C**) on 67NR tumor cells with varying *Map3k1* status after coculture with OT-I splenocytes for 24 hours was determined by flow cytometry. (**D**) Surface expression of MHC-I on tumor cells in **B** and **C** was also measured by immunofluorescence. Scale bar: 50 μm. (**E**) Heatmap showing the significantly downregulated genes in *Map3k1*-mut compared with *Map3k1*-WT tumors in the pathway of antigen processing and presentation via MHC-I. Significance determined as *P* value less than 0.05 and fold change less than 0.8. (**F** and **G**) Comparison of mRNA expression of *Tap1/2* in *MAP3K1*-WT and *MAP3K1*-mut tumors in TCGA (*n* = 481) (**F**) and METABRIC (*n* = 719) (**G**) cohorts. The center line represents the median. Data are mean ± SD (**B** and **C**) (*n* = 3 per group). Statistical analysis: (**B**-**D**) 1-way ANOVA with Tukey’s test. (**F** and **G**) Wilcoxon signed-rank test.

To reveal the mechanism underlying *Map3k1* mutation–mediated immune evasion, we collected *Map3k1*-WT/*Map3k1*-mut tumors isolated from mice, as mentioned above, for RNA-seq. Enrichment analysis on the RNA-seq data demonstrated several immune-related pathways downregulated in *Map3k1*-Mut tumors compared with *Map3k1*-WT tumors, among which the MHC-I mediated antigen presentation pathway (GO: 0019885) might directly explain the findings we had observed (Figure 4A). The downregulation of the MHC-I–mediated antigen presentation pathway in patients with *MAP3K1* mutation was also observed in the METABRIC cohort (Supplemental Figure 5A). To confirm the effect of *Map3k1* on this pathway, we performed the in vitro coculture assay again and collected tumor cells to measure the surface expression of MHC-I and OVA antigen. As a result, we observed less presentation of MHC-I and OVA antigen in *Map3k1*-mut tumor cells compared with the *Map3k1*-WT group using flow cytometry (Figure 4, B and C), in line with the weaker MHC-I fluorescence intensity observed on the surface of *Map3k1*-mut tumor cells (Figure 4D and Supplemental Figure 5B).

We then focused on the downregulated genes in the antigen presentation pathway to determine which step in antigen presentation was suppressed by *Map3k1* mutation (Figure 4E). Among the downregulated genes in the RNA-seq data from mouse tumors, *TAP1* and *TAP2* were also found to have a lower mRNA expression in patients with *MAP3K1* mutation compared with those without in both TCGA and METABRIC cohorts (Figure 4, F and G). However, there was no significant difference in the expression of *HLA-A*, the homologous gene of the other downregulated genes in mouse data, between patients with and without *MAP3K1* mutation (Supplemental Figure 5C). We then confirmed the downregulation of TAP1/2 expression in *Map3k1*-mut cancer cells compared with *Map3k1*-WT cancer cells in the coculture system by quantitative PCR with reverse transcription (RT–qPCR) analysis and Western blot assay (Supplemental Figure 6, A and B). These results displayed that *MAP3K1* mutation could inhibit *TAP1* and *TAP2* expression to disturb the endogenous antigen presentation.

To further determine whether *Map3k1* regulated surface MHC-I expression via regulation of *Tap1*/*2* expression, we performed a rescue assay by overexpressing *Tap1*/*2 in Map3k1*-mut tumor cells. In the coculture system, we found that transient overexpression of *Tap1*/*2* could efficiently rescue the downregulation of MHC-I expression in the tumor cell surface and that the reduced proportions of IFN-γ^+^CD8^+^ and TNF-α^+^CD8^+^ T cells in OT-I splenocytes induced by *Map3k1* mutation (Supplemental Figure 6, C and D and Supplemental Figure 6, E–H), which validated the key role of *Tap1/2* in the modulation of tumor antigen presentation impacted by *Map3k1*.

### Map3k1 mutation destabilizes Tap1/2 mRNAs.

**Figure 5:**
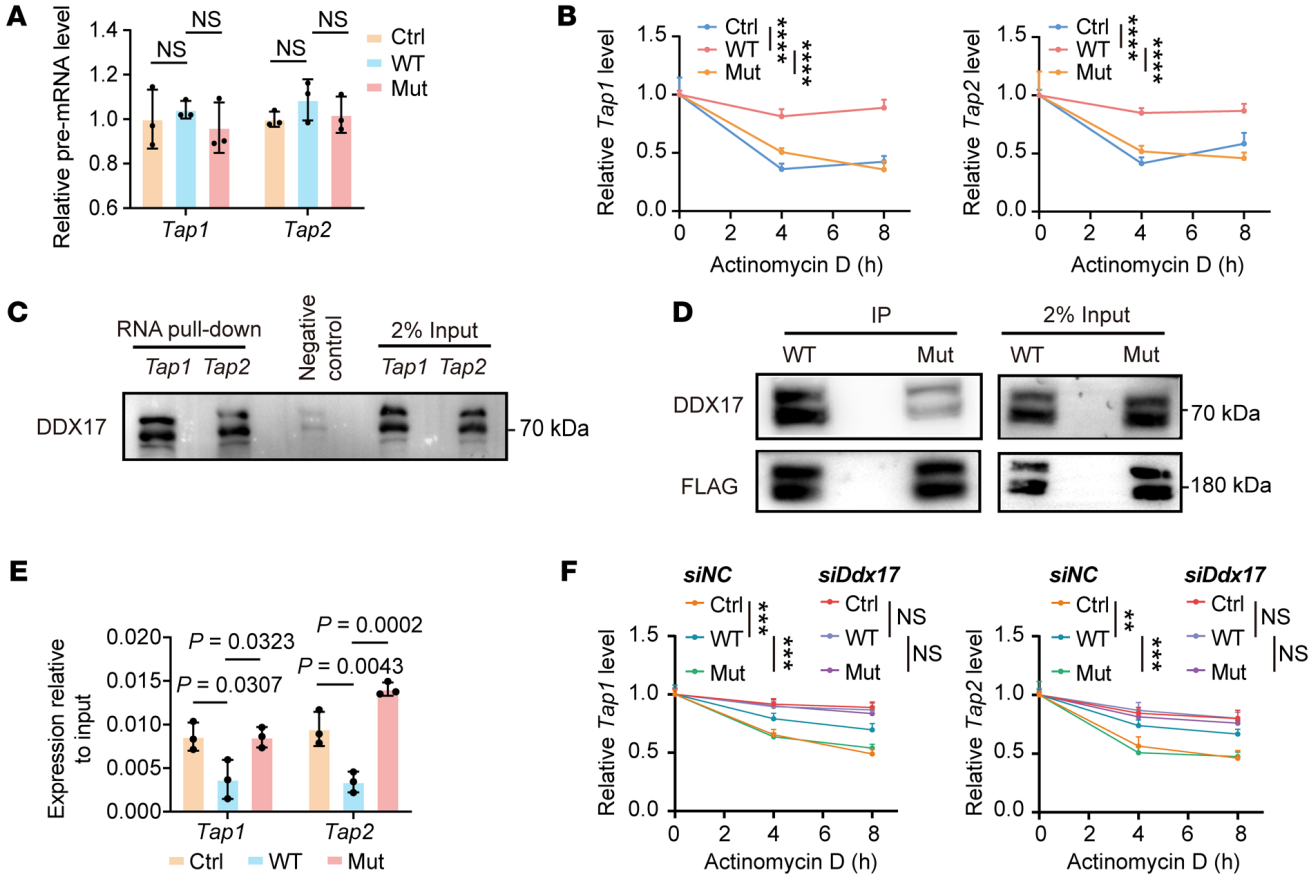
*Map3k1* mutation promotes *Tap1/2* RNA degradation. (**A**) The premature mRNA level of *Tap1/2* in 67NR-OVA cells with varying *Map3k1* status in coculture was measured by RT-qPCR. (**B**) 67NR-OVA cells with varying *Map3k1* status were cocultured with OT-I splenocytes for 24 hours and then treated with actinomycin D at a dose of 10 μg/mL. Tumor cells were collected at the indicated time points to perform RT-qPCR to test the RNA level of *Tap1/2*. (**C**) RNA pull-down assay was performed to examine the binding of *Tap1/2* mRNAs to DDX17 in 67NR-OVA cells in coculture. (**D**) IP assay was performed to examine and compare the binding of *Map3k1*-WT and *Map3k1*-mut to DDX17 in 67NR-OVA cells in coculture. (**E**) 67NR-OVA cells with varying *Map3k1* status in coculture were collected and RNA immunoprecipitation (RIP) assay was performed to extract the RNA binding to DDX17. RT-qPCR was then performed to measure the RNA levels of *Tap1/2*. (**F**) 67NR-OVA cells with varying *Map3k1* status were transiently transfected with small interfering RNA targeting *Ddx17* (*siDdx17*) or its control RNA (*siNC*). A day after transfection, tumor cells were cocultured with OT-I splenocytes for 24 hours and then treated with actinomycin D at a dose of 10 μg/mL. Tumor cells were collected at the indicated time points and RT-qPCR was performed to test the RNA level of *Tap1/2*. Data are mean ± SD (**A**, **B**, **E**, and **F**) (*n* = 3 per group). Statistical analysis: (**A** and **E**) 1-way ANOVA with Tukey’s test. (**B** and **F**) 2-way ANOVA with Tukey’s test. Significance in the RNA degradation experiments (**B** and **F**) is annotated as *P < 0.05, **P < 0.01, ***P < 0.001, ****P < 0.0001.

We then investigated how *Map3k1* mutation downregulated *Tap1*/*2* expression. Initially, we explored whether *Map3k1* could regulate the transcription level of *Tap1*/*2*. We performed the RT-qPCR analysis but observed no significant difference in the premature *Tap1*/*2* expression between *Map3k1*-WT and *Map3k1*-mut tumor cells in coculture with splenocytes from OT-I mice, indicating that *Map3k1* might regulate *Tap1*/*2* expression in a posttranscriptional manner (Figure 5A). We then tested the stability of *Tap1*/*2* mRNA in tumor cells in the coculture system. In summary, we treated the coculture system with 10 μg/ml actinomycin D and collected tumor cells at 4 and 8 hours after treatment for RT-qPCR analysis. We observed that, compared with *Map3k1*-WT, *Map3k1*-mut could significantly promote *Tap1*/*2* mRNA degradation in tumor cells (Figure 5B). Considering that MEKK1 is not an RNA binding protein (RBP), we speculated that MEKK1 could destabilize *Tap1*/*2* mRNAs through another molecule, which could bind and degrade *Tap1*/*2* mRNAs (Supplemental Figure 7A).

We therefore performed RNA pull-down and IP assays to identify RBPs that could bind *Tap1*/*2* mRNA and proteins that interacted with MEKK1, respectively. We focused on the intersection of the proteins identified in the 2 assays (Supplemental Figure 7B), among which DEAD box helicase 17 (DDX17) triggered our interest. DDX17 is an RNA helicase that had been reported to be widely involved in RNA metabolism (19, 20). We confirmed the direct binding of *Tap1*/*2* RNA to DDX17 by RNA pull-down (Figure 5C) and we also observed less binding of *Map3k1*-mut to DDX17 compared with *Map3k1*-WT by IP assay (Figure 5D). In addition, we performed an RNA immunoprecipitation (RIP) assay to compare the ability of DDX17 to bind *Tap1*/*2* mRNAs in *Map3k1*-WT and *Map3k1*-Mut tumor cells in the coculture system. As a result, we found that, compared with *Map3k1*-WT, *Map3k1*-mut significantly attenuated the binding of DDX17 to *Tap1*/*2* mRNAs (Figure 5E). In summary, these results support our speculation that the binding of *Map3k1*-WT to DDX17 could weaken the ability of DDX17 to bind and degrade *Tap1*/*2* mRNAs. *Map3k1*-mut lost this effect, leading to promoted degradation of *Tap1*/*2* mRNAs in *Map3k1*-mut compared to *Map3k1*-WT tumor cells.

We then performed rescue assays using a small interfering RNA targeting *Ddx17* (*siDdx17*). Tumor cells with varying *Map3k1* mutation status in the coculture system were transfected with *siDdx17* and the actinomycin D treatment assay was performed again. We found that silencing *Ddx17* abolished the promoting effect of *Map3k1*-mut on the *Tap1*/*2* mRNA degradation (Figure 5F). Consistently, we observed comparable mRNA and protein levels of TAP1/2 between *Map3k1*-WT and *Map3k1*-mut tumor cells in the coculture system after silencing *Ddx17* (Supplemental Figure 8, A and B). Moreover, we confirmed that silencing *Ddx17* could increase surface MHC-I and OVA antigen expression of *Map3k1*-mut tumor cells, as measured by both immunofluorescence (Supplemental Figure 8C) and flow cytometry (Supplemental Figure 8, D and E, and Supplemental Figure 9, A–D), and thus enhanced the activation of CD8^+^ T cells in the coculture system. All these results indicated that MEKK1 mutation could inhibit its binding to DDX17 and thus promote DDX17-mediated degradation of *Tap1*/*2* mRNAs.

### Tyramine augments the efficacy of immunotherapy in HR^+^/HER2^–^ breast cancer.

As demonstrated above, *Map3k1* mutation could reshape the immune microenvironment by reducing the surface MHC-I expression to promote tumor growth. We then attempted to hunt for strategies that can efficiently reverse the surface MHC-I downregulation. We noticed that recently, postbiotics released from *L*. *paracasei* were reported to induce a significant increase in surface MHC-I expression on several tumor types, including breast cancer (21). We therefore tested the efficacy of the metabolites in the postbiotic mixture to upregulate surface MHC-I in our cell lines. As a result, we found that the metabolites tyramine (Tyra) and 3-hydroxy-3-methylglutarate (3-Hydr) could significantly upregulate surface MHC-I in both 67NR and EMT6 cell lines, while sucrose could not (Supplemental Figure 10, A–F). Considering the commonality of tyramine in daily life and therefore its better potential for clinical transformation, we then applied Tyra into the in vitro coculture system and found that Tyra could efficiently reverse the downregulation of surface MHC-I and OVA antigen in *Map3k1*-mut tumor cells, thus enhancing the activation of CD8^+^ T cells in the coculture system (Supplemental Figure 10, G–J). At last, we investigated whether Tyra could augment the efficacy of anti-PD-1 immunotherapy in the mouse models.

**Figure 6:**
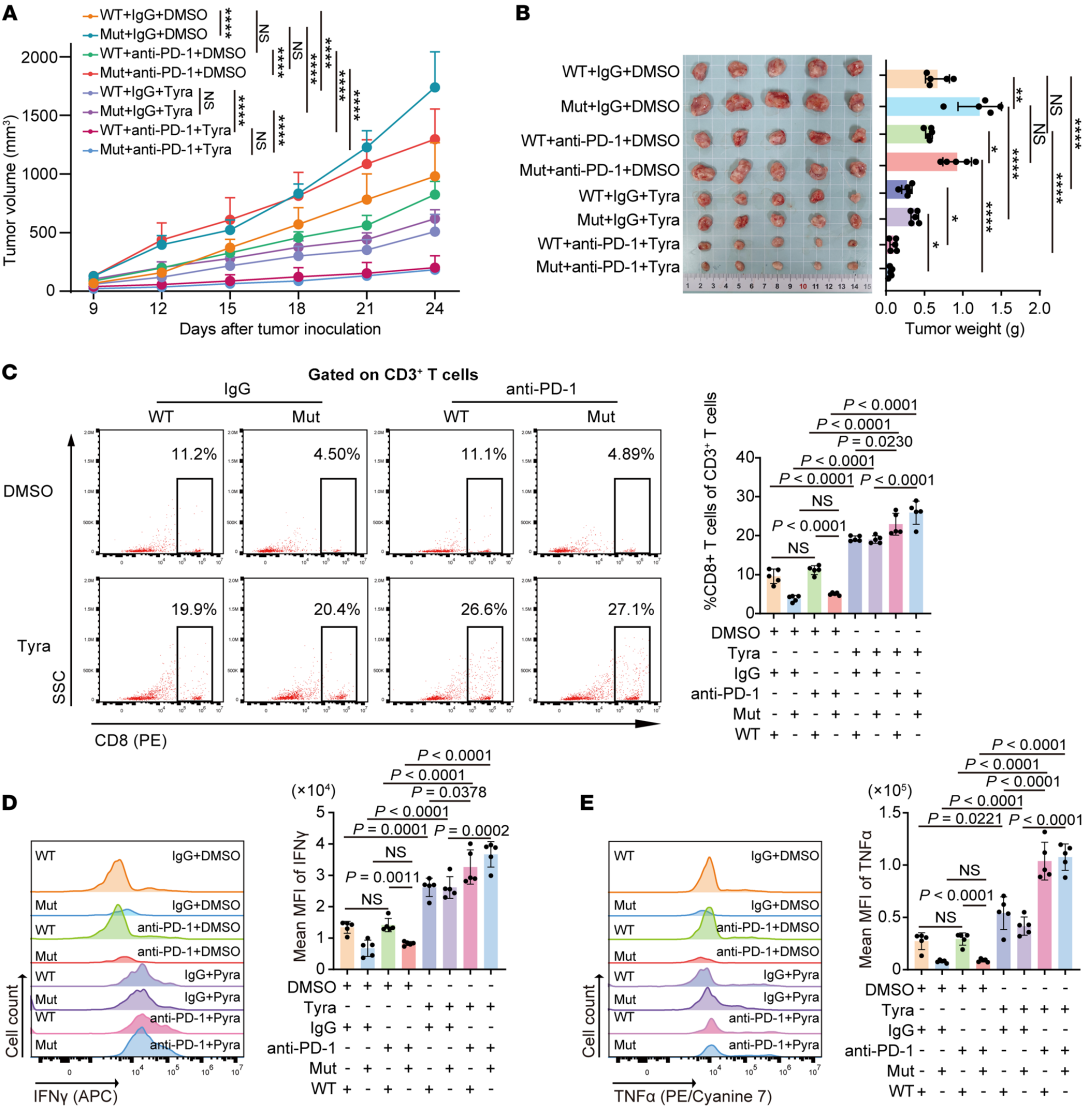
Tyramine augments the efficacy of immunotherapy in HR^+^/HER2^–^ breast cancer. (**A** and **B**) 67NR cells expressing *Map3k1*-WT or *Map3k1*-mut were orthotopically injected into BALB/c mice. Once tumors formed, mice were randomly assigned to receive tyramine (Tyra) or DMSO in combination with anti–PD-1 or its isotype control (IgG). Tumor growth curves (**A**) and tumor weights with the images (**B**) are shown here. (**C**) Representative flow cytometry data of CD8^+^ T cell infiltration in tumor tissues. (**D** and **E**) MFI of IFN-γ (**D**) and TNF-α (**E**) in CD8^+^ T cells in the tumor tissues. Data are mean ± SD (**A**–**E**) (*n* = 5 per group). Statistical analysis: (**A**) 2-way ANOVA with Tukey’s test; (**B**–**E**) 1-way ANOVA with Tukey’s test. Tyra, tyramine. HR, hormone receptor; HER2, human epidermal growth factor receptor 2.

We found that anti-PD-1 treatment alone achieved limited success in suppressing tumor growth, while the combination of Tyra and anti–PD-1 treatment induced strong tumor growth inhibition (Figure 6, A and B). Moreover, the analysis of flow cytometry on tumor tissues demonstrated that the combination of Tyra and immunotherapy significantly increased the infiltration level (Figure 6C) and promoted the activation (Figure 6, D and E) of CD8^+^ T cells. Taken together, the results indicate that the combination of postbiotic Tyra with PD-1 inhibitor could be a therapeutic strategy for the *MAP3K1* mutation subgroup in HR^+^/HER2^–^ breast cancers.

## Discussion

Our multi-omics analysis of a large cohort of patients revealed that *MAP3K1* mutation was associated with an immunosuppressed microenvironment in HR^+^/HER2^–^ breast cancers. Administration of Tyra could reverse the downregulation of tumor antigen presentation caused by *MAP3K1* mutation, thus promoting CD8^+^ T cell–mediated antitumor immunity and enhancing the efficacy of anti–PD-1 immunotherapy in HR^+^/HER2^–^ breast tumors in vivo.

Currently, certain progress has been made for immunotherapy in treating TNBC (22, 23) and the FDA has approved pembrolizumab for this disease. However, immunotherapy has not yet been widely applied to HR^+^/HER2^–^ breast cancer, which accounts for two-thirds of all breast cancers. A key reason for this limited application is the lack of understanding regarding the factors contributing to the unresponsiveness of HR^+^/HER2^–^ breast cancer to immunotherapy. Here, we systematically revealed the immunological heterogeneity of a large cohort of patients with HR^+^/HER2^–^ breast cancer (*n* = 351). This cohort had a nontreated baseline, and, therefore, there is no treatment influence on the immune status. We discovered that the immunosuppressive tumor microenvironment was associated with a high prevalence of *MAP3K1* mutation, suggesting that *MAP3K1* plays an important role in mediating resistance to immunotherapy.

*MAP3K1* is one of the most frequently mutated cancer genes in luminal breast cancer (24). Previous studies have demonstrated the functions of *MAP3K1* in promoting cell survival, invasion, migration, and other cellular functions. (15–17, 25–27). It should also be noted that *MAP3K1* is one of the nodes in the MAPK pathway. Although there is evidence that MAPK pathway blockade, such as MEK inhibitors, could enhance antitumor immune responses (28, 29), little is known about whether and how *MAP3K1* itself modulate s the tumor microenvironment. There are also bioinformatics analyses indicating an association between *MAP3K1* mutation and an immunosuppressive tumor microenvironment (30–32), but evidence from experimental data is scarce and the underlying mechanisms remain elusive. In our study, we reported for the first time that *Map3k1* mutation could promote tumor growth through attenuating CD8^+^ T cell–mediated antitumor immunity by downregulating tumor antigen presentation.

Although downregulation of MHC-I on tumor cells has been discovered early as one of the means of immune escape (33–35), no clear answer is yet available for how to overcome this obstacle in clinic (36). IFN-γ is known to induce strong increases in surface MHC-I expression through the JAK-STAT pathway (37). However, the opposite effects and immunosuppressive functions (such as upregulating PD-L1 expression) of IFN-γ has limited its clinical application (38). Recently, several novel strategies have been identified to specifically upregulate MHC-I expression, including gene perturbations targeting TRAF3 (39) and microbial metabolites (21), among others. Here, we demonstrated that the postbiotic Tyra, the metabolite of *Lactobacillus paracasei*, could efficiently upregulate surface MHC-I expression and augment the efficacy of immunotherapy in HR^+^/HER2^–^ breast cancer. Given that postbiotics have been proven safe for human use, there is potential for rapidly translating Tyra into clinical practice, particularly in combination with existing immunotherapies.

Our study has some limitations. Since the prevalence of *MAP3K1* mutation in HR^+^/HER2^–^ breast cancer is less than 20%, the immunosuppressive tumor microenvironment cannot be entirely attributed to *MAP3K1* mutation. Whether other genes play a role in the immune escape of HR^+^/HER2^–^ breast cancer, and whether *MAP3K1* can modulate the immunity in other cancer types remains unknown. In terms of the mechanism, we observed that *Map3k1*-WT could bind to DDX17 and thus weaken its ability to bind and degrade *Tap1/2* mRNAs. However, the specific binding sites between DDX17 and the other 2 molecules remains elusive. Moreover, how the binding of *Map3k1*-WT reduces the degradation ability of DDX17, i.e., whether through inducing a conformational change of DDX17 or just be a competitively binding, still needs to be further explored. In conclusion, our study demonstrated the ability of *MAP3K1* to modulate the tumor microenvironment via regulation of tumor antigen presentation and highlighted the clinical potential of the postbiotic Tyra to enhance immunotherapy efficacy in HR^+^/HER2^–^ breast cancer.

## Methods

### Sex as a biological variable

Our study exclusively examined female mice because the disease modeled is only relevant in females.

### Study cohorts

Our study included HR^+^/HER2^–^ breast cancer patients from a previously described cohort (CBCGA; 100% female, average age = 53 ± 11 years; 351 patients with RNA-seq data, among whom 315 had whole exome sequencing [WES] data). (11) TCGA (a total of 1,097 patients, 1.1% male and 98.9% female, average age = 58 ± 13 years; including 550 patients with HR^+^/HER2^–^ breast cancer with RNA-seq data, 0.9% male and 99.1% female, average age = 58 ± 13 years) and METABRIC (a total of 1,985 patients, 100% female, average age = 61 ± 13 years; including 1,222 patients with HR^+^/HER2^–^ breast cancer with RNA-seq data, 100% female, average age = 63 ± 12 years) cohorts were from the cBioPortal website (https://www.cbioportal.org/) and analyzed for external validation.

### Cell lines

The mammary carcinoma cell line 67NR (gifted by Y. Kang Lab) was cultured in RPMI-1640 with 10% FBS, 2 mM glutamine, and 100 U penicillin/0.1 mg/mL streptomycin. EMT6 cell line (Cat CRL-2755) and human embryonic kidney cell line HEK293T (Cat CRL-3216) were purchased from the American Type Culture Collection and were cultured in DMEM containing 10% FBS, 2 mM glutamine and 100 U penicillin/0.mg/mL streptomycin. Mycoplasma contamination was regularly monitored for all cell lines by the standard PCR method. Mouse splenocytes freshly isolated from OT-I mice were cultured in RPMI-1640 with 10% FBS, 1% HEPES, 1% sodium pyruvate, 0.05 mM β-mercaptoethanol, and 100 U penicillin/0.1 mg/mL streptomycin.

### Animal studies

#### General.

6–8-week-old female BALB/c mice were purchased from Charles River and housed at Shanghai Laboratory Animal Center under SPF conditions. Mice were housed in individually ventilated and pathogen-free cages with free access to water and a standard chow diet under the following conditions: 20–22°C ambient temperature, 60% ± 10% humidity, and 12 hour light/darkness cycles. Mouse health status was monitored through culture, serum, and microscopic examination.

#### Tumor models.

BALB/c mice were orthotopically injected with 1 × 10^6^ 67NR or EMT6 murine cells with varying *Map3k1* status. Once the tumors formed, tumor size was measured every 3 days using a caliper. Tumor volume (mm^3^) was calculated by the following formula: 0.5 × Length × Width^2^.

#### Mouse treatments.

Mice were treated with 400 μg Tyra in 40 μl DMSO or 40 μl DMSO alone in combination with anti-PD-1 (200 mg; BioXCell, Cat BE0146) or its isotype control (200 mg; BioXCell, Cat BE0089) upon tumors formation. For CD8^+^ T cell depletion experiments, mice were treated weekly with 200 μg of CD8a depletion antibody (InvivoMAb anti-mouse CD8, BioXcell, Cat BE0061) or its isotype control (InVivoMAb rat IgG2b isotype control, BioXcell, Cat BE0090) for 3 weeks by intraperitoneal injection.

### Method details

#### Estimation of immune and stromal cell numbers and unsupervised clustering.

The Microenvironment Cell Populations-counter (MCP-counter) method was performed using the R package “MCPcounter” to quantify the absolute abundance of 8 immune and 2 stromal cell populations from RNA-seq data (40). Based on the MCP-counter results, we used R package “NbClust” to determine the best number of clusters (41) and then classified the samples using PAM clustering (“pam” function in R package “cluster”) into 2 clusters (42).

#### Mutation atlas.

The WES data of each cohort was used to perform the mutation-related analyses. The gene mutation rate in the ICold and IHot tumors was compared using a 2-tailed Fisher’s exact test to identify the ICold-specific mutated genes. “lollipopPlot” function in R package “maftools” was used to generate the *MAP3K1* mutation atlas.

#### Extrapolation.

We extrapolated our immune subtypes into the immunotherapy arm of I-SPY2 HR^+^/HER2^–^ breast cancer cohort using “pamr” package. Genes with SD ranking in the top 80% were used for extrapolation. Before using the ‘‘pamr.train’’ and ‘‘pamr.predict’’ functions, the expression data was normalized using “scale” function in R.

#### CIBERSORT.

The abundance of CD8^+^ T cells in tumors was calculated the Cell-type Identification by Estimating Relative Subsets of RNA Transcripts (CIBERSORT) tool (https://cibersort.stanford.edu/) and then compared between the *MAP3K1*-WT and *MAP3K1*-mut tumors (43).

#### Flow cytometry analysis.

After in vivo experiments, mouse tumors were fully and quickly chopped using scalpel and digested in RPMI-1640 + 5% FBS + 2.5% HEPES + 100 U penicillin/0.1 mg/mL streptomycin + 200 U/mL collagenase III (Sigma-Aldrich) at 37°C with rotation for 60–90 minutes. Samples were then passed through a 70 μm cell strainer and then lysed with red blood cell lysis buffer (Solarbio, R1010) for 5 minutes at room temperature. Then, the single-cell suspensions were washed in Cell Staining Buffer (BioLegend, Cat 420201) and were stained with Zombie Red Fixable Viability Kit (BioLegend, Cat 423110; 1:1,000 in DMSO) at 4°C for 10 minutes, and then incubated with a monoclonal antibody against CD16/32 (BioLegend, Cat 101320) at 4°C for 10 minutes, and then incubated with the indicated flow antibodies at 4°C for 30 minutes. All the flow cytometry antibodies are included in the antibody list in Supplemental Table 5 and are as follows: CD45 (clone 30-F11, BioLegend, Cat 103107), CD3 (clone 17A2, BioLegend, Cat 100222), CD8a (clone 53-6.7, BioLegend, Cat 100708), Ep-CAM (clone G8.8, BioLegend, Cat 118240), H-2K^b^/H-2D^b^ (clone 28-8-6, BioLegend, Cat 114619), IFNγ (clone XMG1.2, BioLegend, Cat 505810), and TNFα (clone MP6-XT22, BioLegend, Cat 506324). For intracellular staining of mouse IFN-γ and TNF-α, samples were stimulated with cocktail (Invitrogen, Cat 00-4975-03) for 4 hours, fixed with fixation buffer (Invitrogen, Cat 00-8222-49), permeabilized with Permeabilization buffer (Invitrogen, Cat 00-8333-56), and then incubated with surface antibodies and finally intracellular antibodies to IFN-γ and TNF-α for 30 minutes at 4°C. A CytoFLEX software (Version 2.4.0.28, Beckman Coulter) and FlowJo software (Version 10.8.1, TreeStar) were used for further analyses.

#### IHC staining.

Sections were incubated with rabbit-anti-mouse CD8 antibody (Servicebio, Cat GB15068, 1:400) overnight, stained with goat anti-rat IgG Ab (Servicebio, Cat GB21303, 1:300) at room temperature for 50 minutes, and then counterstained with hematoxylin.

#### Enrichment analysis.

For mouse RNA-seq data, the student’s *t* test was used to identify the Differentially Expressed Genes (DEGs), and GO enrichment analysis was performed using “enrichGO” function in R package “clusterProfile” based on DEGs. For human transcriptomic data in METABRIC cohort, “limma” package was used to perform the DEG analysis, and Gene set enrichment analysis (GSEA) was performed using the “gseGO” function.

#### Viral production and cell transfection.

The CRISPR/Cas9 plasmids (MiaoLingBio, Cat P51417) were cut using Esp3I enzyme (Thermo Fisher Scientific, Cat FD0454), guided DNA targeting murine *Map3k1* (sequence listed in Supplemental Table 2) was then ligated to the plasmids. Overexpression plasmids (MiaoLingBio, Cat P14038) encoding full-length *Map3k1* (WT) and kinase domain-truncated form (Mut) were generated through ECoRI and BamHI double enzyme cleavage. The authenticity was verified by sequencing. For coculture assays, OVA-expression plasmids (MiaoLingBio, Cat P53920) were also used for transfection. All plasmids were packaged into viruses using HEK293T cells along with helper plasmids psPAX2 and pMD2.G and then transfected into cells. Infected cells were selected using puromycin (for KO transfection) and blasticidin (for *Map3k1* overexpression transfection). Cells were cultured for several generations between different transfections to restore cell viability.

#### Splenocyte isolation.

OT-I mice were killed by carbon dioxide and spleens were collected on a cell strainer, crushed, and rinsed with serum-free RPMI-1640 medium. The filtrate was collected into 50-ml conical tubes and spun down at 130*g* for 5 minutes. Supernatant were removed and then the samples lysed with red blood cell lysis buffer (Solarbio, R1010) for 5 minutes at room temperature. Samples were then spun down and resuspended in culture medium, counted and plated into the coculture system.

#### Immune and tumor cell coculture assay.

67NR-OVA/EMT6-OVA tumor cells with varying *Map3k1* status were seeded into plates in the culture medium listed above. Splenocytes were added into the coculture system once the tumor cells reached 50% confluency at a 10:1 ratio (immune cells-to-tumor cells). The culture medium and cells were collected for further experiments after 24 hours of coculture.

#### ELISA.

Culture medium was collected from OT-I and tumor cell coculture system and the concentration of IFN-γ and TNF-α was measure by the Mouse IFN-γ (Abcam, Cat ab282274) and TNF-α (Abcam, Cat ab208348) ELISA Kits according to the manufacturer’s instructions.

#### RT–qPCR analyses.

The FastPure Cell/Tissue Total RNA Isolation Kit V2 (Nanjing Vazyme Biotech, Cat RC112-01) was used to isolate the total RNA from tumor tissues or cells according to the manufacturer’s instructions. RNA was reverse transcribed into cDNA with the HiScript II 1st Strand cDNA Synthesis Kit (Vazyme, China). Taq Pro Universal SYBR qPCR Master Mix (Vazyme, China) was used to perform the RT-qPCR assay. All the primers and their sequences are listed in Supplemental Table 2. All experiments were repeated for at least 3 times.

#### IP and mass spectrometry.

67NR-OVA tumors cells with varying *Map3k1* status were collected for IP assay after coculture with OT-I splenocytes for 24 hours. Samples were lysed with IP/CoIP Kit (Absin, Cat abs955) according to the manufacturer’s instructions. Samples were incubated with FLAG antibodies (Cell Signaling Technology, Cat 70586) at 4°C for 5 hours and then denatured in 5 × loading buffer containing SDS in boiling water for 10 minutes. Boiled samples were used for Western blot or mass spectrometry. For mass spectrometry, we decolorized and washed the gels containing samples to make it transparent. Gels were freeze-dried and samples disulfide bonds were reduced by dithiothreitol, alkylated, and then enzymatically hydrolyzed. The peptide segment was extracted and dried in vacuum. At last, samples were desalinated and the supernatant was added to the sample bottle for mass spectrometry (Q-extraction) detection.

#### RIP.

After 24 hours of coculture, 67NR-OVA tumors cells with varying *Map3k1* status were collected for RIP assay using an RIP kit (Fitgene, Cat FI88707) according to the manufacturer’s instructions. In brief, 2 × 10^7^ tumor cells were lysed in 500 μL of RIP lysis buffer for 30 minutes on ice. Samples were then centrifuged at 18,000*g* for 15 minutes at 4°C. A total of 30 μL of supernatant was collected as protein input and another 30 μL of supernatant was collected as RNA input. The remaining supernatant was incubated with DDX17 antibodies (Proteintech, Cat 19910-1-AP) at room temperature for 2 hours. 40 μL washed resin was added into each sample and incubated at 4°C for 2 hours. Samples were washed 3 times using lysis buffer. At the last wash, 100 μL of the supernatant was collected for Western blot (tube A), and the rest 400 μL of the supernatant was collected for RNA isolation (tube B). Both tubes were centrifuged at 18,000*g* for 5 minutes at 4°C, and supernatant was removed. For tube A, 20 μL 2 × loading buffer was added and samples were boiled for 3 minutes and then stored at –80°C. For tube B, 40 μL of elution buffer was added and samples were eluted at room temperature for 10 minutes, and then centrifuged at 18,000*g* for 5 minutes, supernatant was collected and RNA was isolated using the method listed above.

#### RNA pull down.

A RNA pull–down assay was then performed using a PureBinding RNA-Protein pull-down Kit (GENESEED, Cat P0202). In brief, a total of 1 × 10^7^ tumor cells were lysed in Capture Buffer supplemented with sufficient protease inhibitors and RNase inhibitors at 4°C for 10 minutes. Samples were centrifuged at 21,000*g* for 10 minutes at 4°C, 50 μL of supernatant were collected as input, and the rest of supernatant was used for further experiment. In the meantime, 50μL of Streptavidin Magnetic Beads for each sample was washed by Wash Buffer in a new 1.5 mL centrifuge tube and then added with 50 pmol *Tap1*/*2* RNA probe, or 2 μL of negative RNA probe (contained in the kit). *Tap1*/*2* mRNAs were obtained through in vitro transcription using Ribo RNAmax-T7 biotin labeled in vitro transcription kit (RIBOBIO, Cat C11002-1) and negative RNA probe was included in the RNA pull–down kit. The mix was incubated at 4°C with rotation for 30 minutes and then washed by Wash Buffer. The supernatant was then divided equally into the mix, and was incubated at 4°C with rotation for 1 hour. Samples were washed by wash buffer 5 times. At the last wash, supernatant was removed and the protein was used for Western blot or mass spectrometry.

#### Western blotting.

Total protein in cells was extracted and lysed in modified RIPA Lysis Buffer (Beyotime Biotech, Cat P0013C) added with protease inhibitors (Beyotime Biotech, Cat ST506). Protein concentrations were measured by BCA protein assay reagent (Yeasen, Cat 20201ES90). Cellular proteins were separated by 10% SDS-PAGE and then transferred to PVDF membranes (Millipore, Cat IPVH00010). The membranes were incubated with the indicated primary antibodies, and the corresponding signals were detected by an enhanced chemiluminescent substrate kit (Yeasen, 36208ES76). Proteins with similar molecular weights (TAP1 and TAP2) were detected by 2 repeated experiments and incubation separately.

#### Colony-formation assay.

A total of 1,000 67NR/EMT6 tumor cells with varying *Map3k1* status were plated into 6-well plates in the culture medium listed above. After 7 days of culture, we removed the culture medium and rinsed the pates with PBS 3 times. Samples were then stained with Crystal Violet Staining Solution (Beyotime Biotech, Cat C0121) at room temperature in darkness for 1 hour. Staining solution was then removed and samples were washed by PBS 3 times and images were captured.

#### Immunofluorescence.

A total of 1 × 10^5^ 67NR/EMT6 tumor cells with varying *Map3k1* status with or without the indicated treatment were plated on sterile slides in 24-well plates. Samples were fixed in 4% PFA for 10 minutes, blocked in 5% BSA (Solarbio, Cat A8010) for 1 hour, incubated with MHC-I Antibody (Affinity, Ca# DF8558) at 4°C overnight. Samples were washed with PBS 3 times before each step. The next day, samples were washed with PBS and then incubated with 594-conjugated Goat anti-Rabbit IgG (H+L) (ABclonal, Cat AS039, 1:200, diluted by 0.1% BSA) at room temperature for 1 hour. Samples were then washed again and were dripped with Antifade Mounting Medium with DAPI (Beyotime Biotech, Cat P0131). Sections were scanned with a Leica SP5 fluorescence confocal microscope 20 × objective (Leica). All images were analyzed using an ImageJ software (Version 2.3.0).

#### Cytotoxicity assays.

The CytoTox 96 Non-Radioactive Cytotoxicity Assay kit (Promega, Cat G1780) was used to measure the cell lysis according to manufacturer’s instructions.

### Statistics

In the multi-omics analysis of HR^+^/HER2^–^ breast cancer cohorts, Kolmogorov-Smirnov normality test was performed to test the data distribution. Continuous variables with nonnormal distribution were compared using the Mann-Whitney Wilcoxon test. Categorical variables were compared using χ^2^ for trend test and Fisher’s exact test. For the in vitro and in vivo experiments, 1-way ANOVA with Tukey’s test was used for 3 or more group comparisons. The time course of tumor volume was compared using 2-way ANOVA with Tukey’s test. All analyses were performed in Graphpad Prism Software (version 8.4.2). A *P* value less than 0.05 was considered significant.

### Study approval

All human tissue samples in the CBCGA cohort were obtained after approval of the research by the FUSCC Ethics Committee, and each patient provided written informed consent for data and tissue use. All animal experiment protocols were approved by the Institutional Animal Care and Use Committee of Shanghai Laboratory Animal Center.

### Data availability

The RNA-seq and WES data of the CBCGA cohort can be viewed in the Genome Sequence Archive (GSA) database under accession codes PRJCA017539 (https://ngdc.cncb.ac.cn/bioproject/browse/PRJCA017539). The TCGA and METABRIC were downloaded from the cBioPortal website (https://www.cbioportal.org/). Mass spectrometry-quantified protein data are provided with this paper (Supplemental Table 3 and 4). The mouse RNA-seq data has been deposited in the NCBI Gene Expression Omnibus (GEO: GSE276527).

This study did not report new original code. Any additional information required to reanalyze the data reported in this paper is available from the corresponding author upon request. Values for all data points in graphs are reported in the Supporting Data Values file.

## Author contributions

KDY designed and supervised the project. YWC and CCL performed the experiments. YWC, LC, YML, YWZ, and XX contributed to data analysis. YWC and CCL wrote the original draft. KDY, ZMS, and CCL reviewed and edited the manuscript. All authors critically revised the draft and approved the final manuscript.

## Supplementary Material

Supplemental data

Unedited blot and gel images

Supplemental tables 1-5

Supporting data values

## Version 1 11/12/2024

In-Press Preview

## Version 2 01/16/2025

Electronic publication
