# Supplementary material for: MAP3K1 mutations confer tumor immune heterogeneity in hormone receptor–positive HER2-negative breast cancer
Source: J Clin Invest. 2024 Nov 12;135(2):e183656. doi: 10.1172/JCI183656 (PMC11735090; doi:10.1172/JCI183656)
Supplement: Supplemental data [file jci-135-183656-s168.pdf]

Supplemental Figures 1-10

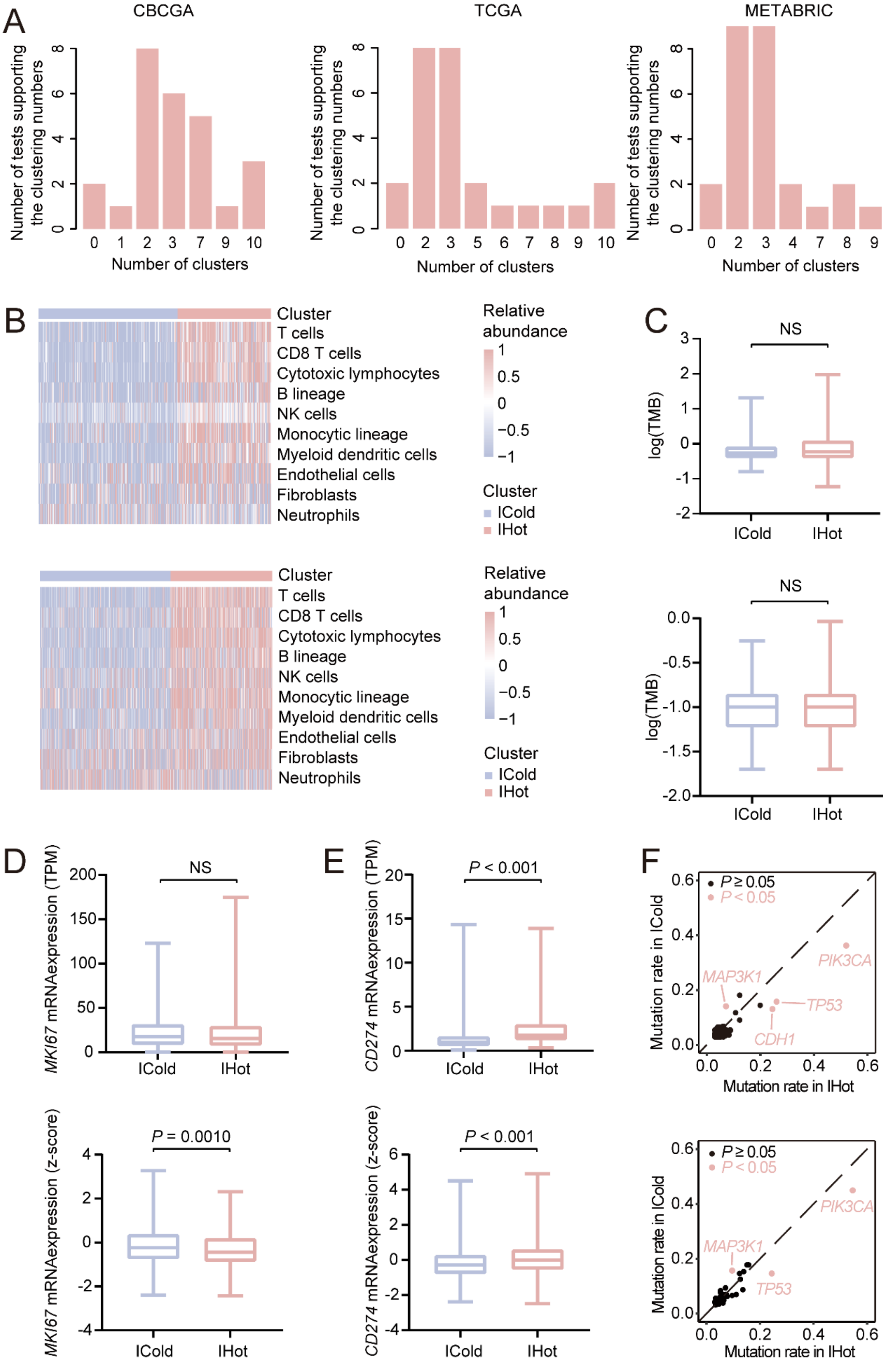

Supplemental Figure 1. ***MAP3K1*** **mutation is closely correlated with immune**

microenvironment heterogeneity in $HR^+/HER2^-$ breast cancer.

(**A**) Number of clusters chosen by 26 criteria provided by "NbClust".

(**B**) Heatmap showing the relative abundance of immune and stromal cells calculated by MCP-Counter in each $HR^+/HER2^-$ breast cancer sample in TCGA (top panel; n = 550) and METABRIC (bottom panel; n = 1222) cohorts. The two immunological subtypes were annotated.

(**C**) Comparison of tumor mutation burden (TMB) of $HR^+/HER2^-$ breast tumors with the ICold and IHot subtypes in TCGA (top panel; n = 475) and METABRIC (bottom panel; n = 611) cohorts. The center line represents the median.

(**D**) Comparison of mRNA expression of *MKI67* in $HR^+/HER2^-$ breast tumors with the ICold and IHot subtypes in TCGA (top panel; n = 543) and METABRIC (bottom panel; n = 1042) cohorts. The center line represents the median.

(**E**) Comparison of mRNA expression of *CD274* in $HR^+/HER2^-$ breast tumors with the ICold and IHot subtypes in TCGA (top panel; n = 543) and METABRIC (bottom panel; n = 1042) cohorts. The center line represents the median.

(**F**) Mutation prevalence of cancer-related genes in TCGA (top panel; n = 475) and METABRIC (bottom panel; n = 611) cohorts.

Statistical analysis: (**C-E**) Wilcoxon signed-rank test; (**F**) Fisher's exact test. ICold, immune cold subtype; IHot, immune hot subtype; HR, hormone receptor; TMB, tumor mutation burden; HER2, human epidermal growth factor receptor 2.

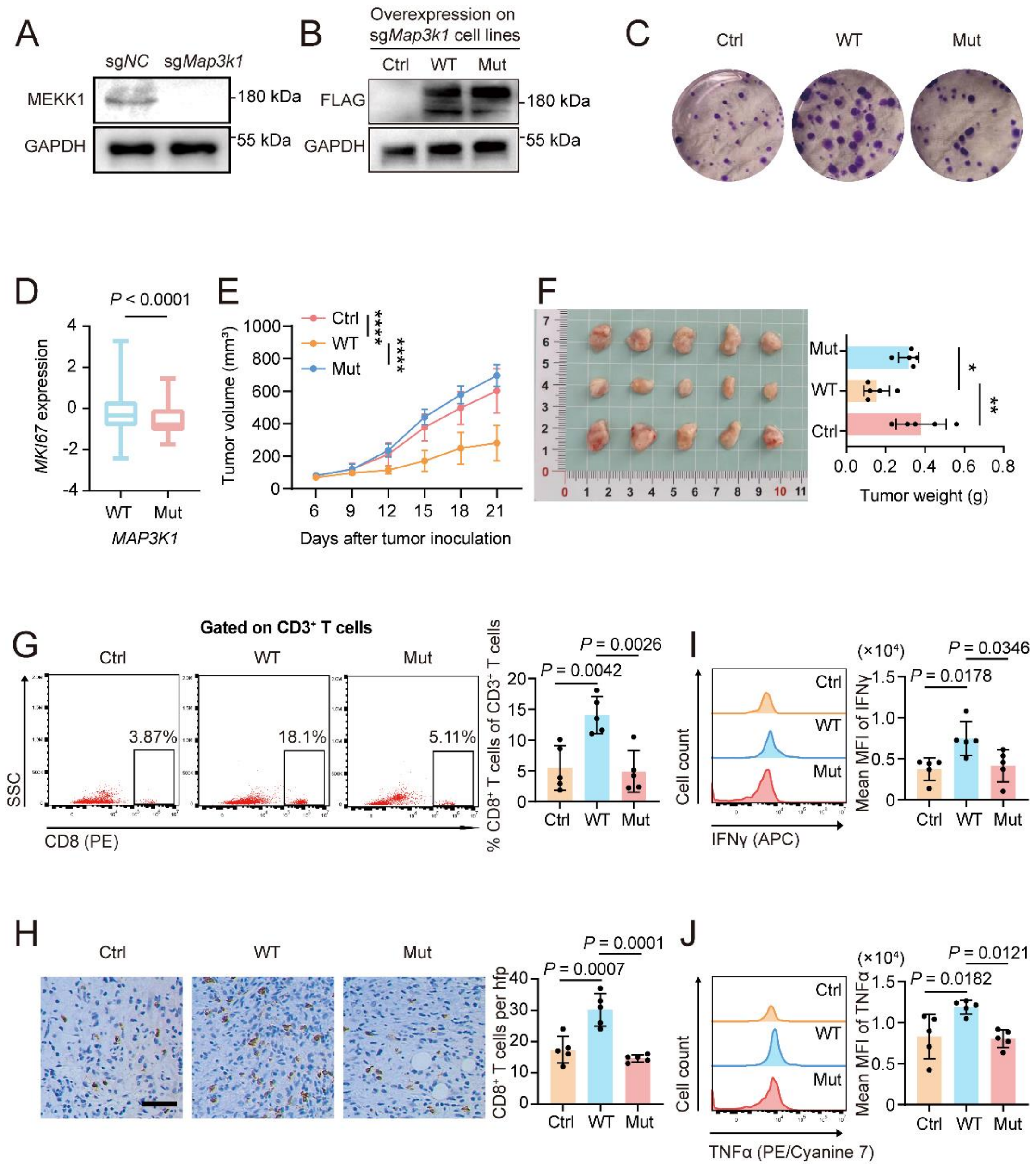

Supplemental Figure 2. *MAP3K1*-mutant tumors evade CD8+ T cell-mediated immunity in vivo. Related to Figure 2.

(**A**) *Map3k1* KO efficacy in 67NR mouse breast cancer cell lines was examined by western blot.

(**B**) *Map3k1* KO 67NR cell lines were stably transfected with *Map3k1*-WT (WT) and *Map3k1*-Mut (Mut) or their control vector (Ctrl). The expression of MEKK1 was confirmed by western blot.

(**C**) Tumor cells in (**B**) were used for colony formation assay and the representative images are shown here.

(**D**) Comparison of the mRNA expression of *MKI67* in *MAP3K1*-WT and *MAP3K1*-Mut tumors (n = 719) in METABRIC cohort. The center line represents the median.

(**E** and **F**) EMT6 mouse breast cancer cells with varying *Map3k1* status were orthotopically injected into BALB/c mice (n = 5 per group). Tumor growth curves (**C**) and tumor weights with the images (**D**) are shown.

(**G**) Representative flow cytometry data of $CD8^+$ T cell infiltration gated on $CD3^+$ T cells in tumor tissues.

(**H**) Representative IHC images of tumor tissues are shown and the numbers of $CD8^+$ T cell are quantified. Scale bar, 50 μm.

(**I** and **J**) MFI of IFNγ (**I**) and TNFα (**J**) in $CD8^+$ T cells in the tumor tissues.

Data are mean ± SD (**E**-**J**) (n = 5 per group). Statistical analysis: (**D**) Wilcoxon signed-rank test; (**E**) two-way ANOVA with Tukey's test; (**F**-**J**) one-way ANOVA with Tukey's test. Significance in tumor growth (**E**) and tumor weight (**F**) data is annotated: *$P < 0.05$, **$P < 0.01$, ***$P < 0.001$, ****$P < 0.0001$.

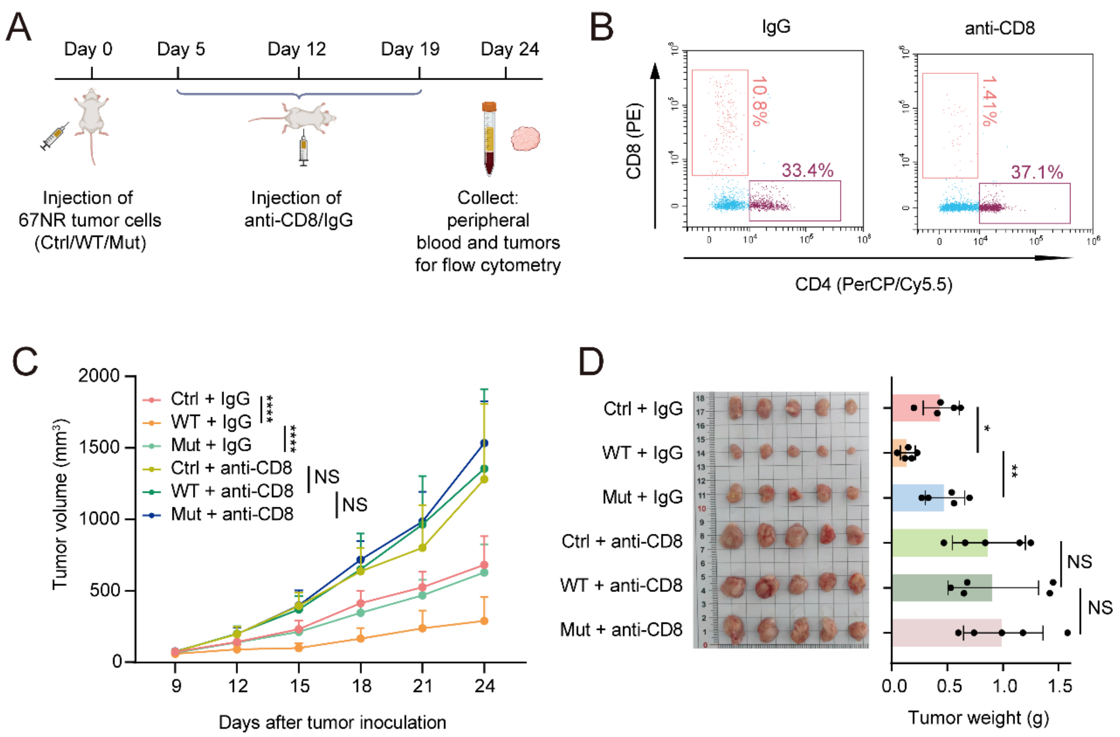

Supplemental Figure 3. *MAP3K1*-mutant tumors evade CD8^{+} T cell-mediated immunity in vivo. Related to Figure 2.

(**A**-**D**) Schematic diagram showing the CD8 neutralizing experiment (**A**). BALB/c mice were orthotopically injected with 67NR cells with varying *Map3k1* status on day 0 and then were injected with a CD8 neutralizing antibody or its isotype control (IgG) at the indicated time points (n = 5 per group). Peripheral blood was collected to examine depletion of CD8 (**B**). Tumor growth curves (**C**) and tumor weights with the images (**D**) are shown.

Data are mean ± SD (**C** and **D**) (n = 5 per group). Statistical analysis: (**C**) two-way ANOVA with Tukey's test; (**D**) one-way ANOVA with Tukey's test. Significance in tumor growth (**C**) and tumor weight (**D**) data is annotated: **P* < 0.05, ***P* < 0.01, ****P* < 0.001, *****P* < 0.0001.

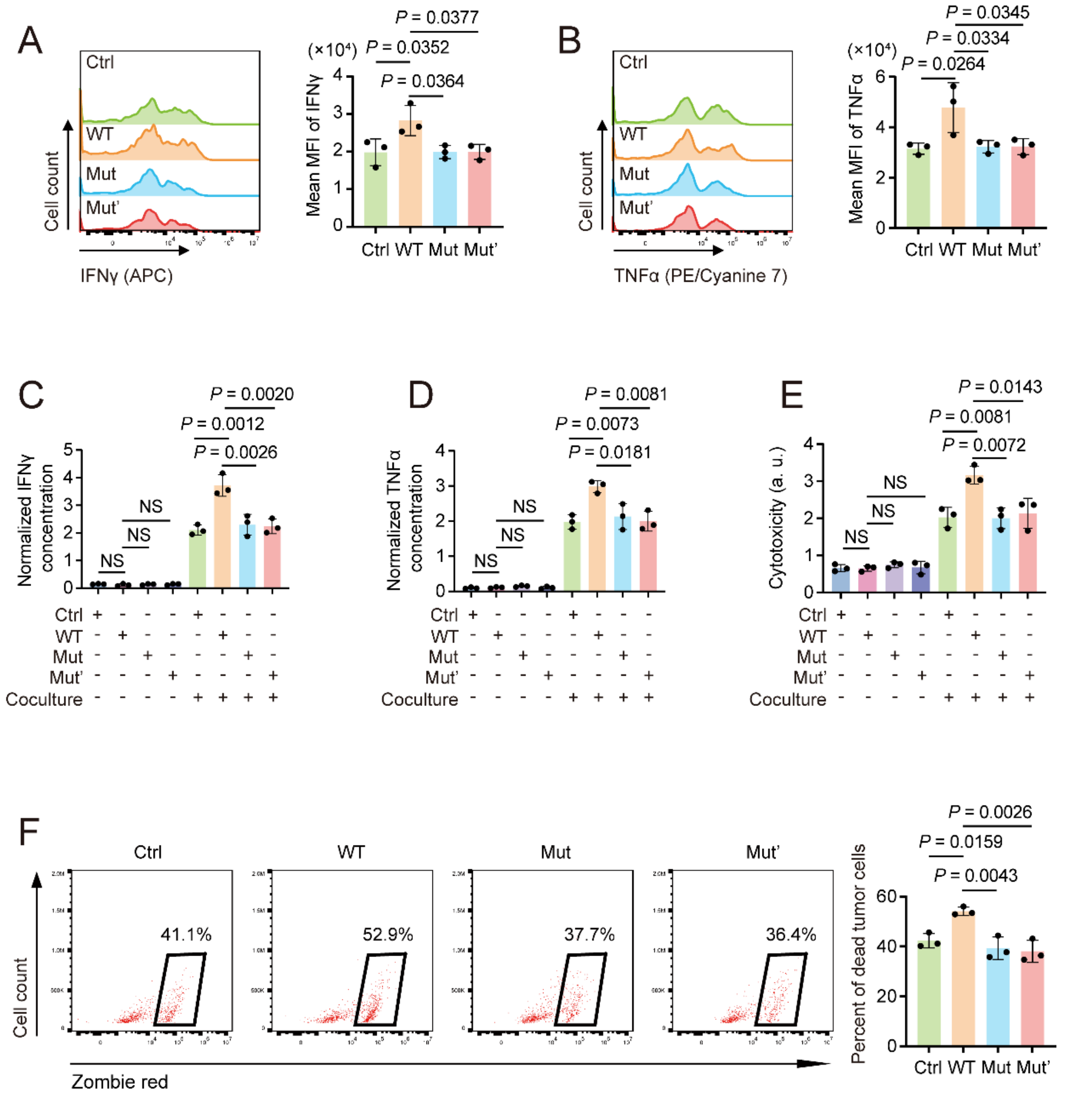

Supplemental Figure 4. ***MAP3K1*** **mutation inhibits CD8^{+} T cells activation in vitro. Related to Figure 3.**

(**A** and **B**) EMT6-OVA cells with varying *Map3k1* status were cocultured with OT-I splenocytes for 24 hours. MFI of IFNγ (**A**) and TNFα (**B**) in CD8^{+} T cells after coculture are shown.

(**C** and **D**) Concentration of cytokines IFNγ (**C**) and TNFα (**D**) in the culture medium of the above EMT6-splenocytes coculture system was measured by ELISA.

(**E**) T cell cytotoxicity in the 67NR-splenocytes coculture system was measured by lactate dehydrogenase (LDH) assay.

(**F**) T cell cytotoxicity was assessed by the percentage of dead cells in EpCAM^{+} tumor cells in the EMT6-splenocytes coculture system.

Data are mean ± SD (**A-F**) (n = 3 per group). Statistical analysis: (**A-F**) one-way

ANOVA with Tukey’s test.

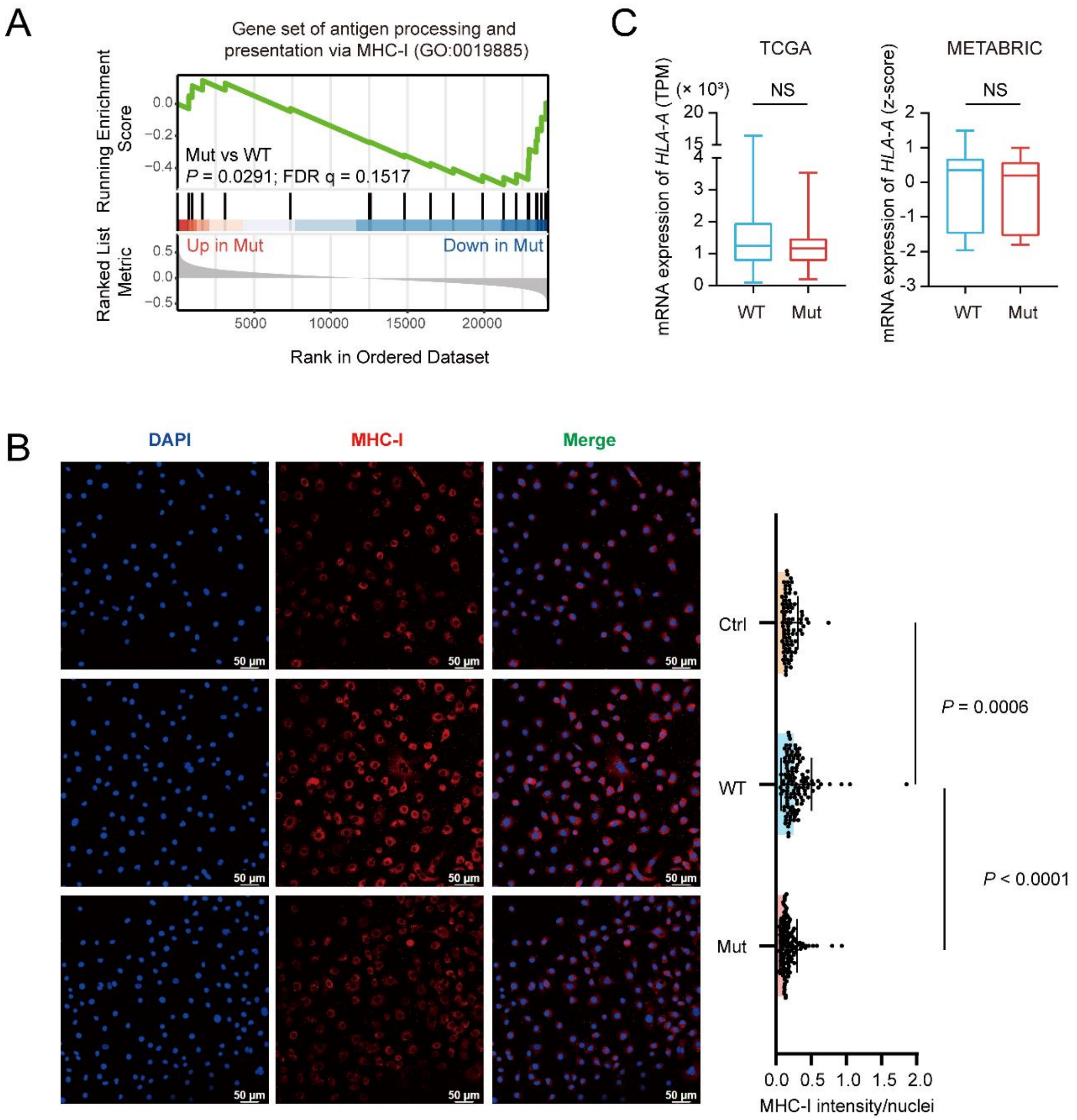

Supplemental Figure 5. ***MAP3K1*** **mutation suppresses tumor antigen presentation. Related to Figure 4.**

(**A**) Representative gene set enrichment analysis plot showing downregulated antigen processing and presentation via MHC-I pathway in patients with *MAP3K1* mutation versus those without in METABRIC cohort. Significance was determined as *P* value less than 0.05 and FDR q value less than 0.25.

(**B**) EMT6 tumor cells with varying *Map3k1* status were cocultured with OT-I splenocytes for 24 hours. Surface expression of MHC-I on tumor cells was measured by immunofluorescence. Scale bar: 50 μm.

(**C**) Comparison of mRNA expression of *HLA-A* in *MAP3K1*-WT and *MAP3K1*-Mut tumors in TCGA (n = 481, left panel) and METABRIC (n = 719, right panel) cohorts.

The center line represents the median.

Statistical analysis: (**C**) Wilcoxon signed-rank test. FDR, false discovery rate.

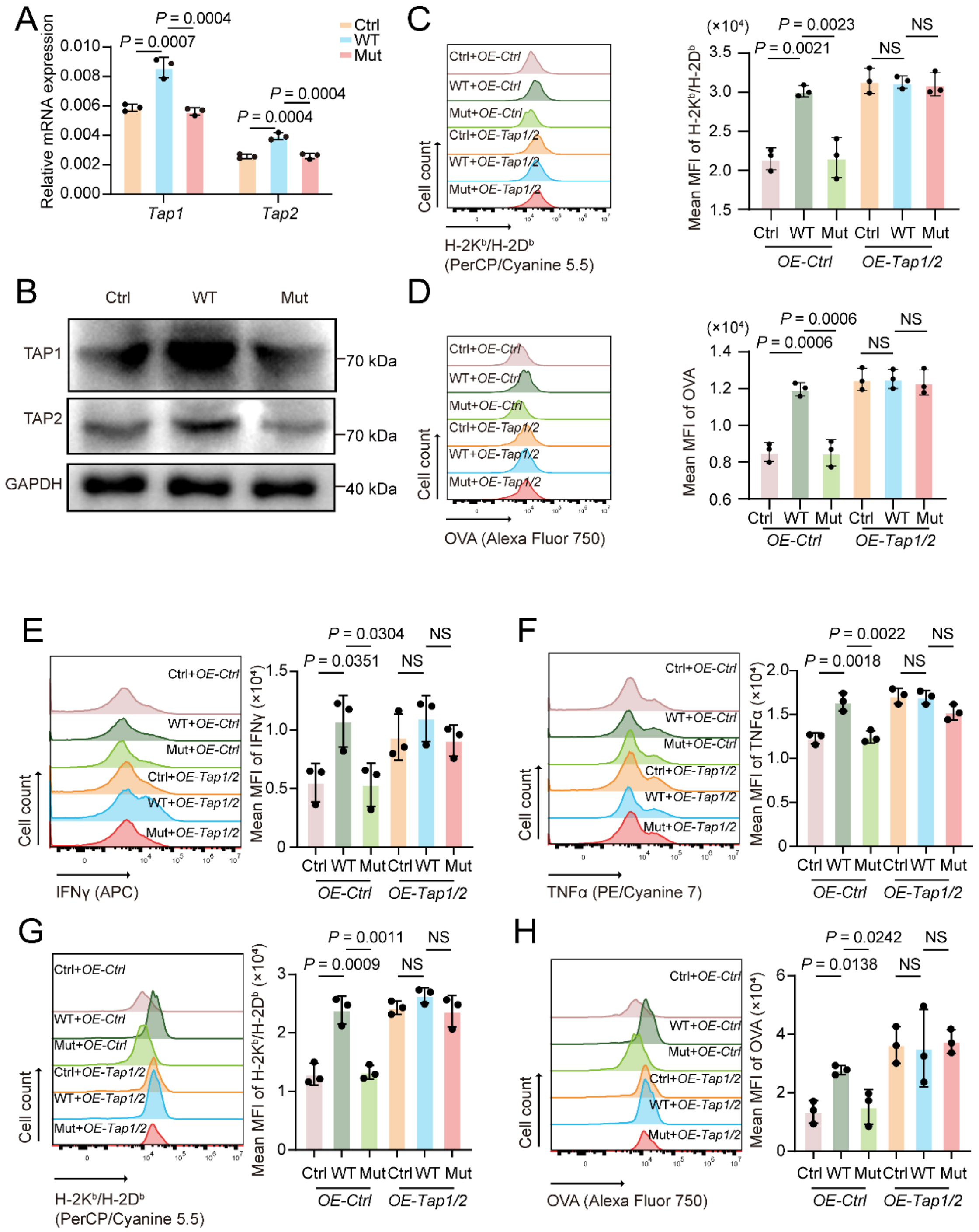

Supplemental Figure 6. *MAP3K1* mutation suppresses tumor antigen presentation. Related to Figure 4.

(**A**) At 24 hours after coculture with OT-I splenocytes, 67NR-OVA cells with varying *Map3k1* status were collected to perform real-time quantitative polymerase chain reaction (RT-qPCR) analysis to test the RNA expression of *Tap1/2*.

(**B**) Tumors cells in (**A**) were also collected for western blot.

(**C** and **D**) OVA-expressing 67NR tumor cells with varying *Map3k1* status were

transiently transfected with *Tap1* and *Tap2* overexpression plasmids (OE-*Tap1/2*) or its control vector (OE-*Ctrl*). One day after transfection, tumor cells were cocultured with OT-I splenocytes for 24 hours. Surface expression of H-2K^b/H-2D^b (**C**) and OVA (**D**) on tumor cells were then measured by flow cytometry.

(**E**-**H**) EMT6-OVA cells with varying *Map3k1* status were transiently transfected with *Tap1* and *Tap2* overexpression plasmids (OE-*Tap1/2*) or its control vector (OE-*Ctrl*). One day after transfection, tumor cells were cocultured with OT-I splenocytes for 24 hours. Expression of IFNγ (**E**) and TNFα (**F**) in CD45$^+$ OT-I splenocytes and surface expression of H-2K^b/H-2D^b (**G**) and OVA (**H**) on CD45$^-$ tumor cells were measured by flow cytometry.

Data are mean ± SD (**A**, **C**, **D**, and **E**-**H**) (n = 3 per group). Statistical analysis: (**A**, **C**, **D**, and **E**-**H**) one-way ANOVA with Tukey's test.

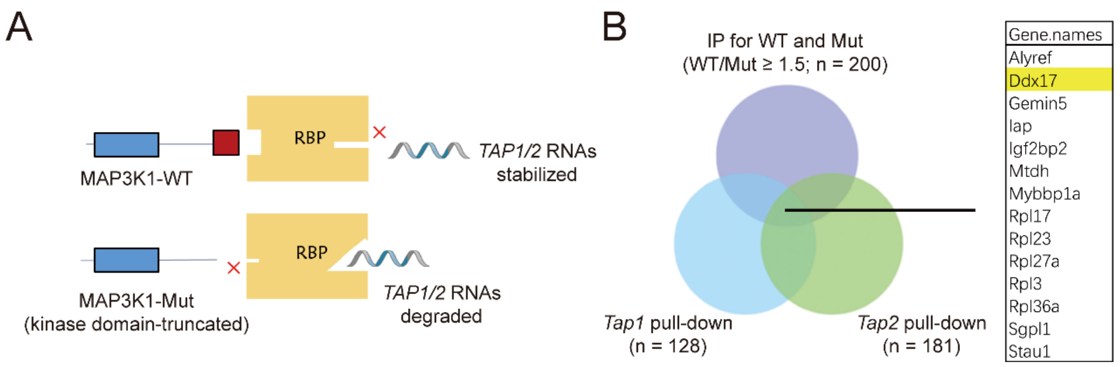

Supplemental Figure 7. *Map3k1* mutation promotes *Tap1/2* RNAs degradation. Related to Figure 5.

(**A**) Schematic diagram of *MAP3K1*-Mut promoting *TAP1/2* RNAs degradation. *MAP3K1*-WT could bind to and induce a conformational change in an RNA binding protein (RBP), and thus weakens the ability of the RBP to bind and degrade *TAP1/2* RNAs. *MAP3K1*-Mut lost this effect, resulting in an increase in *TAP1/2* degradation.

(**B**) IP with mass spectrometry assay was performed to identify proteins that bind more with *Map3k1*-WT than with *Map3k1*-Mut. RNA pull-down with mass spectrometry assay was performed to identify potential RBPs that bind to *Tap1/2* RNAs. The top proteins in intersect of these proteins are listed here.

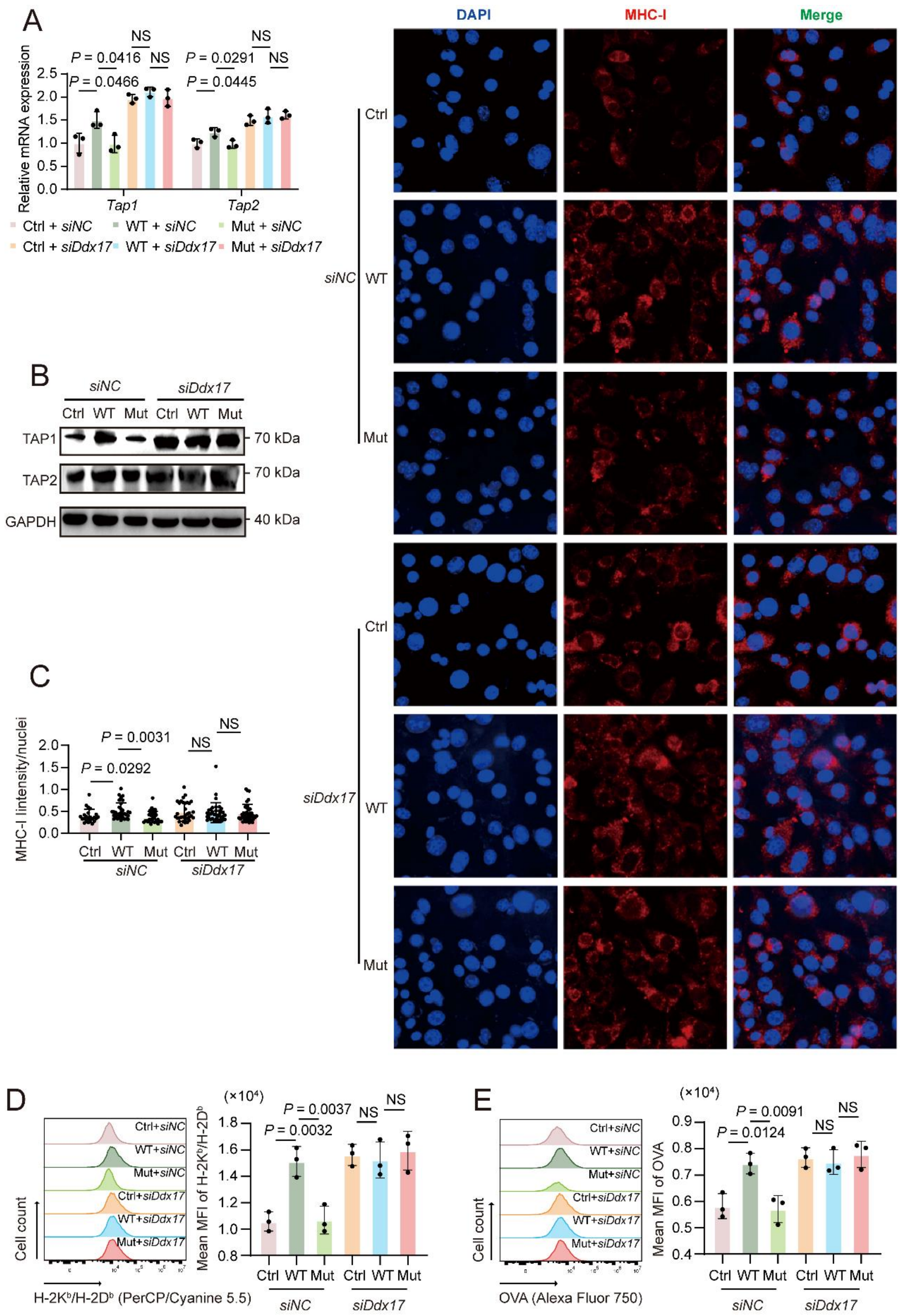

Supplemental Figure 8. *Map3k1* mutation promotes *Tap1/2* RNAs degradation. Related to Figure 5.

(**A**) 67NR-OVA cells with varying *Map3k1* status were transiently transfected with

siDdx17 or *siNC*. One day after transfection, tumor cells were cocultured with OT-I splenocytes for 24 hours. Tumor cells were then collected and RT-qPCR was performed to test the RNA level of *Tap1/2*.

(**B**) Tumor cells in (**A**) were also collected for western blot.

(**C**) Surface expression of MHC-I on tumor cells in (**A**) was measured by immunofluorescence. Scale bar: 50 μm.

(**D** and **E**) Surface expression of $H\text{-}2K^b$/$H\text{-}2D^b$ (**D**) and OVA (**E**) on tumor cells in (**A**) was also measured by flow cytometry.

Data are mean ± SD (**A**, **D**, **E**) (n = 3 per group). Statistical analysis: (**A**, **C**, **D**, **E**) one-way ANOVA with Tukey's test.

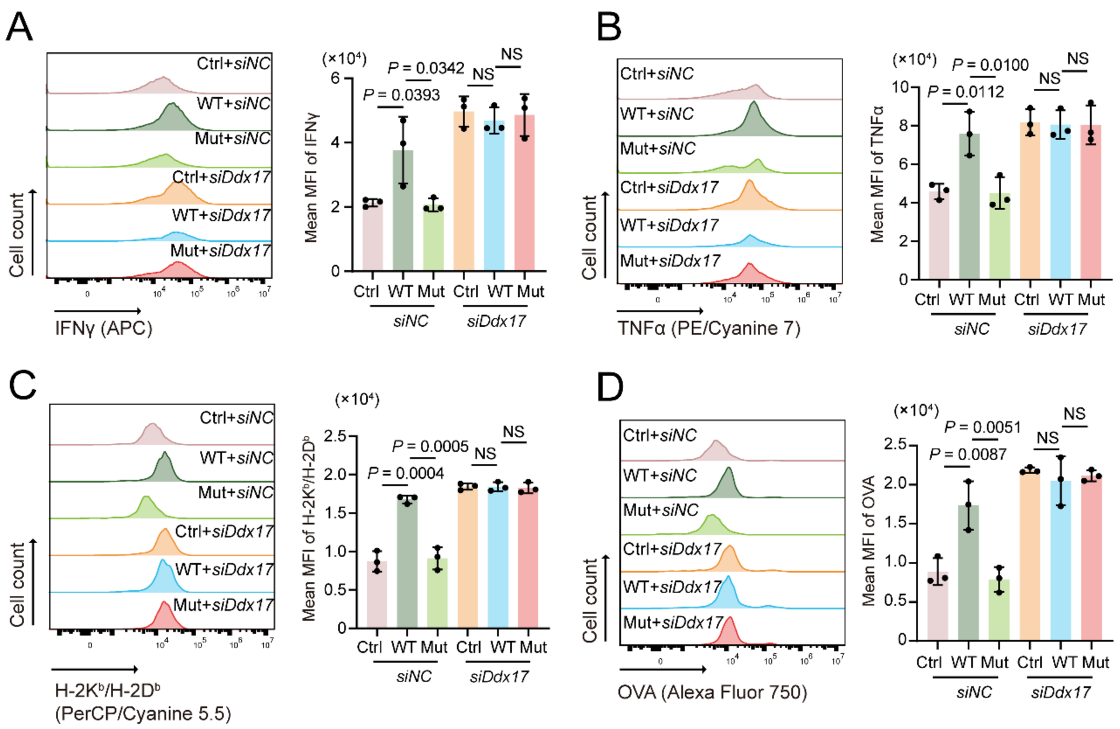

Supplemental Figure 9. *Map3k1* mutation promotes *Tap1/2* RNAs degradation. Related to Figure 5.

(**A-D**) EMT6-OVA cells with varying *Map3k1* status were transiently transfected with small interfering RNA targeting *Ddx17* (*siDdx17*) or its control RNA (*siNC*). One day after transfection, tumor cells were cocultured with OT-I splenocytes for 24 hours. Expression of IFNγ (**A**) and TNFα (**B**) in $CD45^+$ OT-I splenocytes and surface expression of $H\text{-}2K^b/H\text{-}2D^b$ (**C**) and OVA (**D**) on $CD45^-$ tumor cells were measured by flow cytometry.

Data are mean ± SD (**A-D**) (n = 3 per group). Statistical analysis: (**A-D**) one-way ANOVA with Tukey's test. RBP, RNA binding protein.

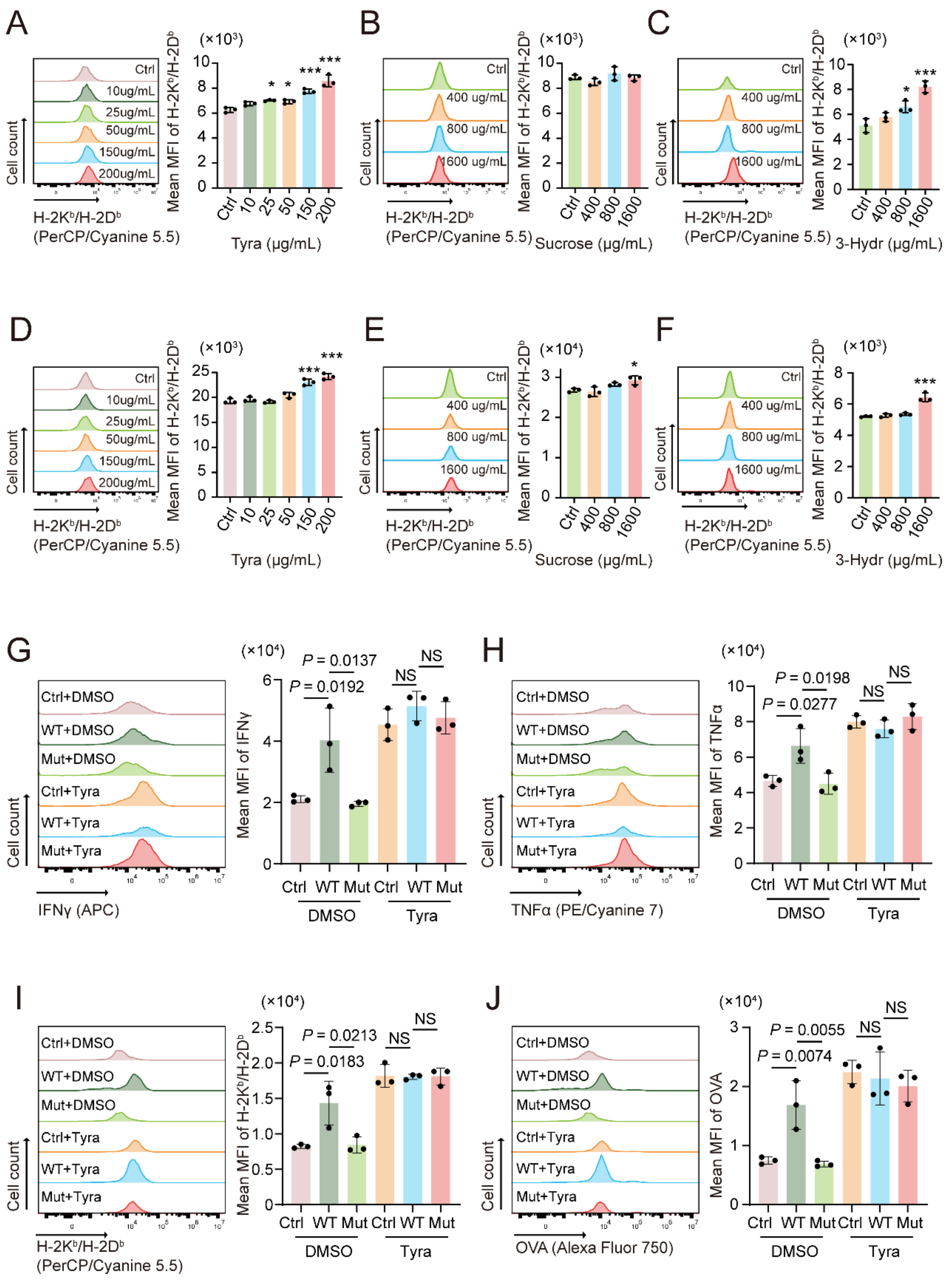

Supplemental Figure 10. Tyramine augments the efficacy of immunotherapy in HR+/HER2- breast cancer. Related to Figure 6.

(**A**-**C**) 67NR cells were incubated with different concentrations of Tyra (**A**), sucrose (**B**), and 3-Hydr (**C**) in vitro. Surface expression of H-2K^b/H-2D^b were measured by flow cytometry.

(**D**-**F**) EMT6 cells were incubated with different concentrations of Tyra (**D**), sucrose (**E**), and 3-Hydr (**F**) in vitro. Surface expression of H-2K^b/H-2D^b were measured by flow cytometry.

(**G**-**J**) 67NR-OVA cells with varying *Map3k1* status were cocultured with OT-I splenocytes in the presence of 200 μg/ml Tyra for 24 hours. Expression of IFNγ (**G**) and TNFα (**H**) in CD45$^+$ OT-I splenocytes and surface expression of H-2K^b/H-2D^b (**I**) and OVA (**J**) on CD45$^-$ tumor cells were measured by flow cytometry.

Data are mean ± SD (**A**-**J**) (n = 3 per group). Statistical analysis: (**A**-**F**) one-way ANOVA with Dunnett's test; (**G**-**J**) one-way ANOVA with Tukey's test. Significance in (**A**-**F**) is annotated: *P < 0.05, **P < 0.01, ***P < 0.001, ****P < 0.0001. Tyra, tyramine. Tyra, tyramine; 3-Hydr, 3-hydroxy-3-methylglutarate. HR, hormone receptor; HER2, human epidermal growth factor receptor 2.
