# Supplementary material for: MAP3K1 mutations confer tumor immune heterogeneity in hormone receptor–positive HER2-negative breast cancer
Source: J Clin Invest. 2024 Nov 12;135(2):e183656. doi: 10.1172/JCI183656 (PMC11735090; doi:10.1172/JCI183656)

MEKK1 for Supplemental Figure 2A

180 kDa

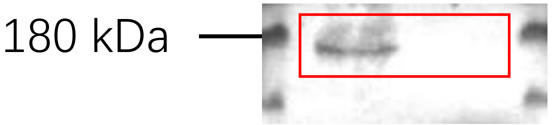

FLAG for Supplemental Figure 2B

180 kDa

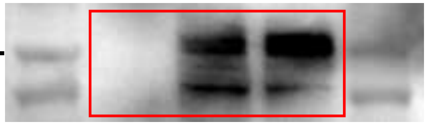

GAPDH for Supplemental Figure 2A

55 kDa

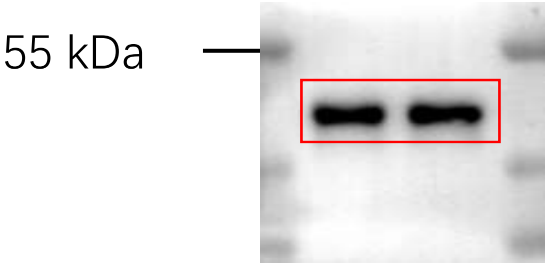

GAPDH for Supplemental Figure 2B

55 kDa

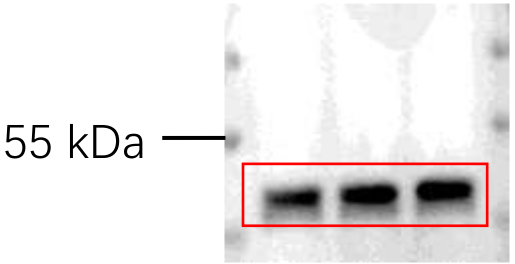

TAP1 for Supplemental Figure 6B

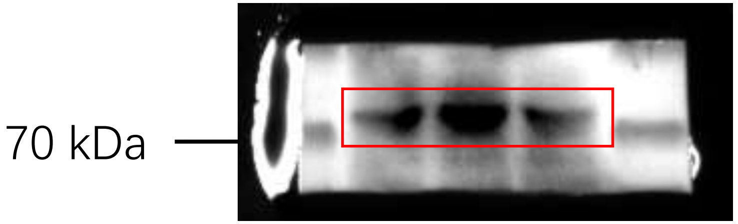

TAP2 for Supplemental Figure 6B

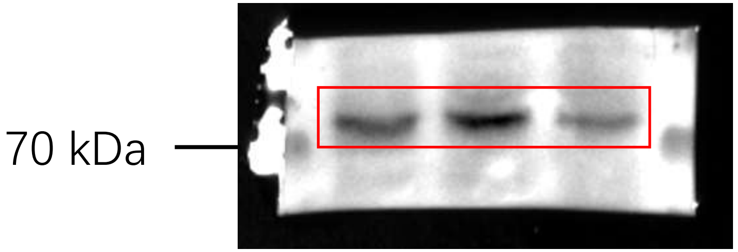

GAPDH for Supplemental Figure 6B

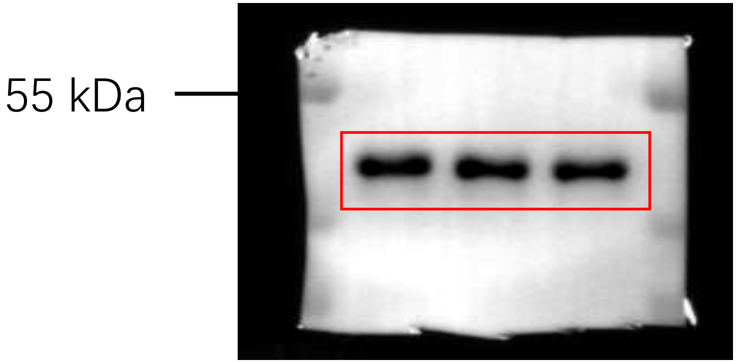

DDX17 for Figure 5C

70 kDa

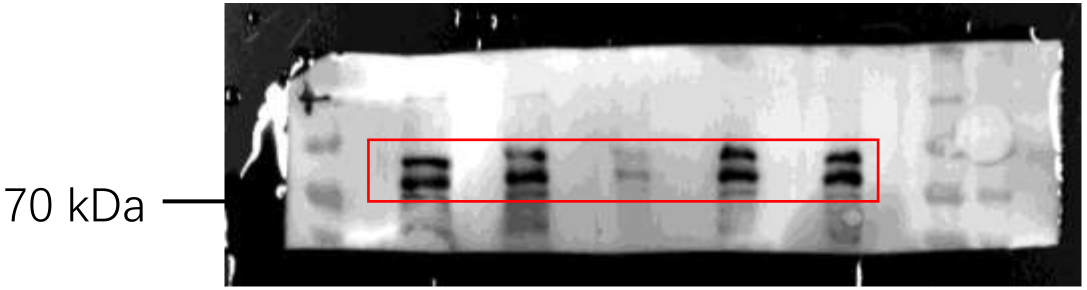

DDX17 for Figure 5D

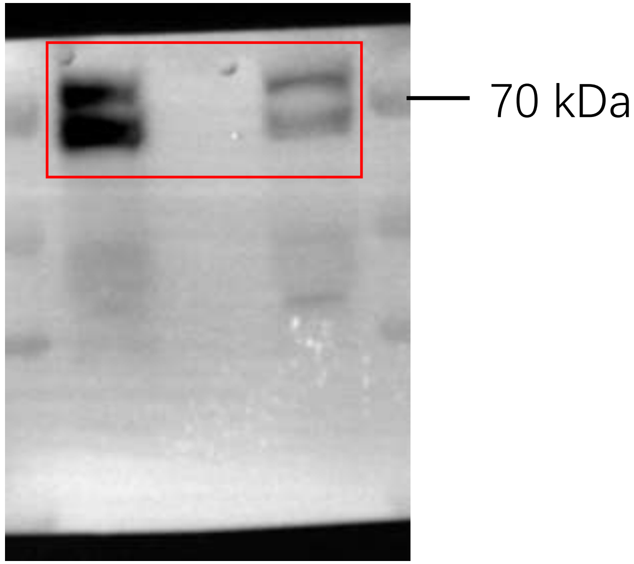

DDX17 for Figure 5D input

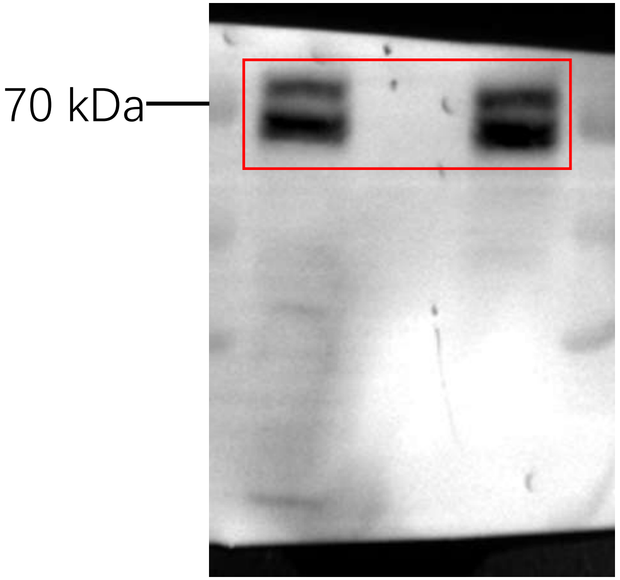

FLAG for Figure 5D

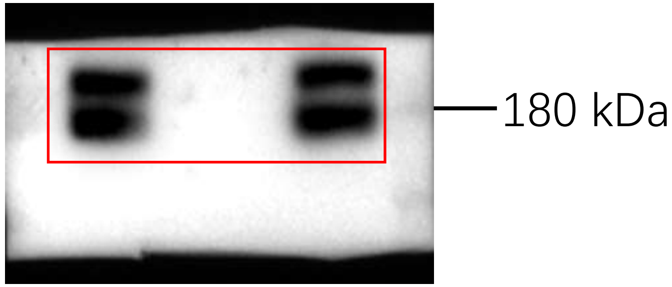

FLAG for Figure 5D input

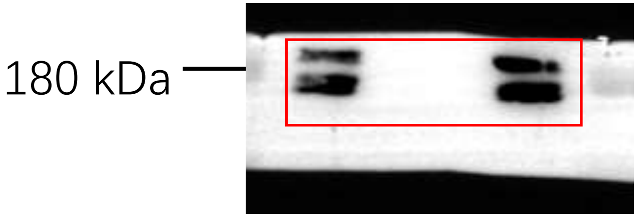

TAP1 for Supplemental Figure 8B

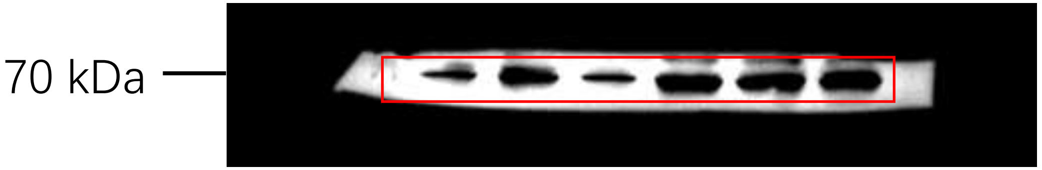

TAP2 for Supplemental Figure 8B

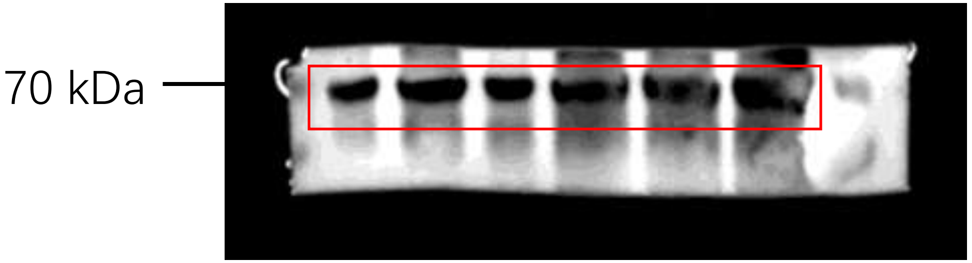

GAPDH for Supplemental Figure 8B

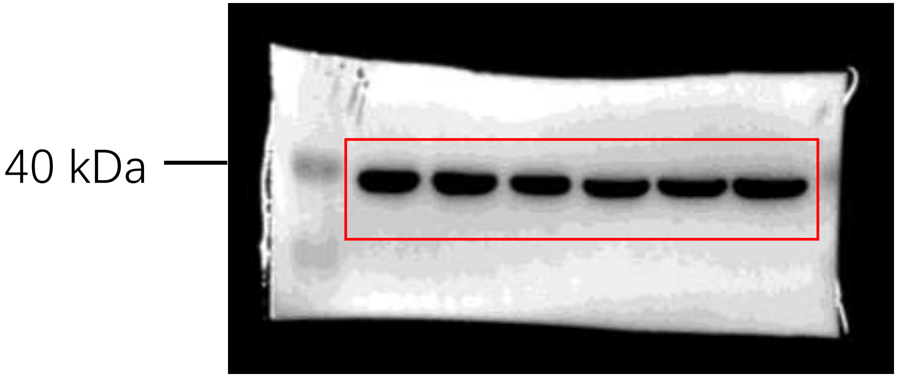

Supplement: Unedited blot and gel images [file jci-135-183656-s169.pdf]
